# Pneumococcal vaccination uptake and missed opportunities for vaccination among Canadian adults: A cross-sectional analysis of the Canadian Longitudinal Study on Aging (CLSA)

**DOI:** 10.1371/journal.pone.0275923

**Published:** 2022-10-14

**Authors:** Giorgia Sulis, Valérie Rodrigue, Christina Wolfson, Jacqueline M. McMillan, Susan A. Kirkland, Melissa K. Andrew, Nicole E. Basta

**Affiliations:** 1 Department of Epidemiology, Biostatistics and Occupational Health, School of Population and Global Health, Faculty of Medicine and Health Sciences, McGill University, Montreal, QC, Canada; 2 Department of Medicine, Faculty of Medicine and Health Sciences, McGill University, Montreal, QC, Canada; 3 Research Institute of the McGill University Health Centre, Montreal, QC, Canada; 4 Department of Medicine, Faculty of Medicine, University of Calgary, Calgary, AB, Canada; 5 O’Brien Institute for Public Health, University of Calgary, Calgary, AB, Canada; 6 Department of Community Health and Epidemiology, Faculty of Medicine, Dalhousie University, Halifax, NS, Canada; 7 Division of Geriatric Medicine, Department of Medicine, Faculty of Medicine, Dalhousie University, Halifax, NS, Canada; Public Health England, UNITED KINGDOM

## Abstract

**Introduction:**

In Canada, pneumococcal vaccination is recommended to all adults aged ≥65 and those <65 who have one or more chronic medical conditions (CMCs). Understanding vaccine uptake and its determinants among eligible groups has important implications for reducing the burden of pneumococcal disease.

**Methods:**

Using data from a large national cohort of Canadian residents aged ≥47 years between 2015–2018, we calculated self-reported pneumococcal vaccine uptake among eligible groups, estimated associations between key factors and non-vaccination, assessed missed opportunities for vaccination (MOV) and examined risk factors for MOV. Adjusted odds ratios (aORs) and 95% confidence intervals (CIs) for relevant associations were estimated through logistic regression.

**Results:**

45.8% (95% CI: 45.2–46.5) of 22,246 participants aged ≥65 and 81.3% (95% CI: 80.5–82.0) of 10,815 individuals aged 47–64 with ≥1 CMC reported never having received a pneumococcal vaccine. Receipt of influenza vaccination in the previous year was associated with the lowest odds of pneumococcal non-vaccination (aOR = 0.14 [95% CI: 0.13–0.15] for older adults and aOR = 0.23 [95% CI: 0.20–0.26] for those aged 47–64 with ≥1 CMC). Pneumococcal vaccine uptake was also more likely in case of contact with a family doctor in the previous year (versus no contact), increased with age and varied widely across provinces. Among individuals recently vaccinated against influenza, 32.6% (95% CI: 31.9–33.4) of those aged ≥65 and 71.1% (95% CI: 69.9–72.3) of those aged 47–64 with ≥1 CMC missed an opportunity to get a pneumococcal vaccine. Among individuals who had contact with a family doctor, 44.8% (95% CI: 44.1–45.5) of those aged ≥65 and 80.4% (95% CI: 79.6–81.2) of those aged 47–64 with ≥1 CMC experienced a MOV.

**Conclusions:**

Pneumococcal vaccine uptake remains suboptimal among at-risk Canadian adults who are eligible for vaccination. Further research is needed to clarify the reasons behind missed opportunities for vaccination and adequately address the main barriers to pneumococcal vaccination.

## Introduction

Pneumonia, together with influenza, is consistently ranked among the top ten causes of death for Canadian adults [1]. Invasive pneumococcal disease (IPD) occurs when *Streptococcus pneumoniae*, the leading bacterial cause of pneumonia, infects a sterile site of the human body (e.g. pleural fluid, blood, cerebrospinal fluid) [2, 3]. In adults, incidence and mortality related to IPD increase with age [4]. National surveillance data from Canada indicate an annual incidence of 21.1 cases per 100,000 among individuals aged ≥60 years in 2017, and–according to provincial data–even higher estimates are reported for older age groups (57.5 cases per 100,000 people for those aged ≥85 years, versus an average estimated incidence rate of 10.8 per 100,000 among all age groups in Ontario between 2010 and 2018) [5, 6].

Due to the higher vulnerability of older adults to pneumococcal disease, the National Advisory Committee on Immunization (NACI) first recommended vaccination of all those aged ≥65 in 1989 with one lifetime dose of the pneumococcal 23-valent polysaccharide vaccine **(**PPV23) [7]. According to this recommendation, which remains in place as of the most recent NACI guidelines (2018) [7], all Canadian provinces have been funding the PPV23 vaccine for their population aged ≥65 years starting in 2001 or earlier [8] (S1 Table). Besides this age-based recommendation, NACI has recommended that select groups of adults aged 18–64 years who have at least one of a range of chronic medical conditions (CMCs) be vaccinated with one lifetime dose of PPV23, a recommendation that has been adopted by provincial vaccination programs [8, 9]. Furthermore, based on current NACI guidance, immunocompromised adults, regardless of age, are recommended to receive one dose of the pneumococcal conjugate 13-valent vaccine (PCV13) eight weeks before receiving one dose of PPV23 vaccine to maximize strain coverage and immune response [9, 10]. Although all provinces have adopted NACI guidelines with respect to pneumococcal vaccination, provincial-level implementation timelines, deployment approaches, and funding schemes vary (S1 Table). Of note, vaccination is offered in a range of settings that vary from province to province, with possible implications for accessibility. Historically, pneumococcal vaccines have been primarily administered in doctor’s offices, but vaccination is becoming increasingly available at pharmacies. In some provinces eligible individuals can get vaccinated in pharmacies at no cost, whereas in other provinces pharmacies may offer this service for a fee (S1 Table). Furthermore, while several high-risk groups are eligible for vaccination in any given province, some of these groups may not be eligible to receive the vaccine at a pharmacy.

Among the more than 90 pneumococcal serotypes that are currently recognized, 15 account for most IPD cases. About half of all serotyped IPD cases among individuals aged ≥65 in Canada are attributable to vaccine-preventable strains [6, 11, 12]. The incidence of vaccine-preventable IPD despite provincial vaccination programs is in part due to the variable vaccine effectiveness, which also tends to decline with time, as well as to low vaccine uptake among eligible groups [13, 14]. A large proportion of those eligible for pneumococcal vaccination in Canada remain unvaccinated, with vaccination coverage in 2020/2021 estimated to be 55% among adults aged ≥65 and 26% among those aged 18–64 with a CMC [15]. The current level of uptake falls short of the 80% coverage target to be achieved by 2025 as established in the National Immunization Strategy [16]. Pneumococcal vaccination rates have also been found to vary greatly in association with diverse individual characteristics, including sex, age, income, and indigenous identity in several surveys among older Canadians [15, 17, 18]. However, most of these surveys assessed a very limited number of variables or were completed in only one location, leaving the need for a pan-Canadian assessment unmet.

A better understanding of the factors associated with non-vaccination among eligible groups is crucial to design and implement strategies that can effectively improve uptake. Given the lack of awareness about the importance of pneumococcal vaccines in general [19], missed opportunities for vaccination (MOV) during clinical encounters, defined by the World Health Organization (WHO) as “any contact made with health services by [an individual] who is eligible for vaccination, but which does not result in the individual receiving […] the vaccine” [20], are of concern, as well, and highlight a potential context in which to improve uptake [17].

Given these concerns, we conducted a cross-sectional analysis using data from the Canadian Longitudinal Study on Aging (CLSA) [21, 22] to address the following objectives: (1) estimate self-reported pneumococcal vaccine uptake and differences in uptake among Canadian adults eligible to be vaccinated (i.e. individuals aged ≥65 and adults aged <65 who had ≥1 CMC); (2) identify factors associated with pneumococcal non-vaccination in these populations; and (3) assess the frequency and determinants of MOV in the same groups.

## Methods

### Study setting and population

The CLSA is a national longitudinal study of 51,338 Canadian residents aged 45–85 years at enrolment (2011–2015), from all ten Canadian provinces [21, 22]. The study includes two cohorts (Comprehensive and Tracking cohorts) that were recruited through three sampling frames and approaches, as detailed elsewhere [21–23]. Three years after the baseline study visit, participants were invited to participate in the follow-up 1 (FU1) survey (2015–2018), and almost 90% (n = 44,817) had available FU1 data. All CLSA survey questionnaires are available on the CLSA website [24]. The core CLSA study has been approved by McMaster University Health Integrated Research Ethics Board and by research ethics boards at all collaborating Canadian institutions. The present study is a secondary analysis of fully deidentified CLSA data which has been approved by McGill University Institutional Review Board (A02-E03-21A (21-02-048)). As such, additional participant consent for this analysis was not required as all CLSA participants provided informed consent during primary data collection to have their de-identified data used in research.

### Data sources

We analysed data collected during the CLSA baseline study visit (conducted between 2011–2015) and the CLSA FU1 visit (conducted between 2015–2018). A detailed description of each survey question and the resulting variables included in our analyses is provided in S2 Table.

### Outcome variable

We investigated self-reported pneumococcal vaccination as the outcome of interest. During the FU1 survey, CLSA participants were asked whether they had had “*a pneumonia shot (pneumococcal vaccination) in [their] life*”. Those who answered “no” were categorized as unvaccinated while those who answered "yes" were categorized as vaccinated. The CLSA combines those who responded “don’t know” with those who refused and failed to answer into a single category, thus preventing us from further examining each of these subgroups; therefore, these responses were collectively classified as missing and excluded from the analysis.

### Sociodemographic variables

To address our objectives, we considered the following sociodemographic characteristics: sex at birth (male or female), age group (47–54, 55–64, 65–74, 75–84, 85 years and older), race (white or any other race), highest education level (less than secondary school graduation, secondary school graduation without post-secondary education, some post-secondary education, post-secondary degree/diploma), annual household income (in Canadian dollars: <$20,000, $20,000 to < $50,000, $50,000 to < $100,000, $100,000 to < $150,000, $150,000 or higher), marital/partner status (single/never married/never lived with a partner, married or living with a partner in a common-law relationship, widowed, divorced/separated), province of residence (ten Canadian provinces), urbanicity of residence (urban or rural). Participants reported their race and education level during the CLSA baseline study visit, whereas all other variables were reported during FU1.

### Variables related to health status and healthcare utilization

We categorized participants as having a chronic medical condition (CMC) if, when prompted during FU1, they self-reported a physician diagnosis of any of the following eight types of conditions, all dichotomized as diagnosed/not diagnosed: cardiovascular disease (including prior heart attack/myocardial infarction, angina or chest pain due to heart disease, hypertension), chronic lung disease (including emphysema, chronic bronchitis, chronic obstructive pulmonary disease, chronic changes in lungs due to smoking, asthma), cerebrovascular disease (including stroke and transient ischemic attack), chronic kidney disease or failure, diabetes mellitus, cancer, and chronic neurologic condition (including dementia, Alzheimer’s disease, Parkinson’s disease, multiple sclerosis). These CMCs are the most frequently associated with increased risk of IPD in older adults and constitute the majority of conditions NACI lists as criteria for eligibility for pneumococcal vaccine in their recommendations [9]. As an indicator of healthcare utilization, we examined whether participants reported any contact with a family doctor in the previous 12 months. We also utilized data about self-reported receipt of influenza vaccination in the previous 12 months (vaccinated/unvaccinated).

### Sample size and missing data

The process utilized to identify CLSA participants who met the inclusion criteria for our analyses is shown in S1 Fig. Among CLSA participants who completed FU1 and met the eligibility criteria for pneumococcal vaccination due to age (≥65 years) or presence of select CMCs according to NACI guidelines, only 5.4% (n = 2,404) of those aged ≥65 and 0% of those aged 47–64 who had ≥1 CMC had unknown self-reported pneumococcal vaccination status. For objective 1, our analyses were thus restricted to 22,246 CLSA participants aged ≥65 years and 10,815 CLSA participants aged 47–64 with ≥1 CMC.

To assess factors associated with non-vaccination (objective 2), we adopted a casewise deletion approach whenever we encountered missing data for any of the other variables included in the models (i.e., resulting in a sample size of 19,742 individuals aged ≥65 years and 10,284 individuals aged 47–64 with ≥1 CMC). The frequency of missing data among participants with known pneumococcal vaccination status was <2.6% for all variables.

To examine MOV (objective 3), we further restricted the analysis to those who reported receiving an influenza vaccine in the previous 12 months (i.e., resulting in a sample size of 15,637 CLSA participants aged ≥65 years and 5,351 aged 47–64 who had ≥1 CMC) or reported having had contact with a family physician in the previous 12 months (i.e. resulting in a sample size of 21,017 individuals aged ≥65 years and 9,927 individuals aged 47–64 who had ≥1 CMC). For our regression models pertaining to MOV, we used casewise deletion as described above for objective 2.

### Statistical analysis

To address objective 1, we calculated the proportion of participants who reported having received a pneumococcal vaccine among individuals aged ≥65 and among those aged 47–64 who had ≥1 CMC. The 95% confidence intervals (CIs) were constructed using a logit transformation of each proportion. We also described the characteristics of these populations at increased risk of IPD and eligible for pneumococcal vaccination by presenting proportions and 95% CIs across strata of key variables.

To identify risk factors for lack of pneumococcal vaccination (objective 2) among each subgroup of interest (i.e., those aged ≥65 years and those aged 47–64 with ≥1 CMC), we used nested logistic regression models to assess the association between each of the independent variables in the following sets and self-reported pneumococcal vaccination (outcome):

- Model 1a and 1b [Sociodemographic Characteristics]: age group (reference: 65–74 years for model 1a investigating those aged ≥65 years and 47–54 years for model 1b investigating those aged 47–64 with ≥1 CMC), sex at birth (reference: female), race (reference: white), highest education level (reference: less than secondary school education), annual household income (reference: <$20,000), marital/partner status (reference: single/never married/never lived with a partner), province of residence (reference: Ontario), and urbanicity of residence (reference: urban).
- Model 2a [CMC Diagnosis and Healthcare Utilization among those aged ≥65 years]: diagnosis of ≥1 CMC among those described previously versus no diagnosis of any of the CMCs listed, contact with a family physician in the past 12 months versus no contact, and self-reported influenza vaccination in the past 12 months versus no vaccination. This model was adjusted for all sociodemographic characteristics included in Model 1a.
- Model 2b [Health Care Utilization among those aged 47–64 years with at least one CMC]: The same structure was used as in Model 2a except the CMC diagnosis variable was omitted.

Based on the results of each model, we reported adjusted odds ratios (aORs) and 95% CIs for the association between each independent variable and the outcome of interest.

To address objective 3, we examined individuals who reported having received an influenza vaccine in the previous 12 months and those who reported having had contact with a family physician in the previous 12 months. Two types of MOV were considered: 1) having received an influenza vaccine in the previous 12 months while being eligible for a pneumococcal vaccine but not receiving pneumococcal vaccine, and 2) having had contact with a family physician in the previous 12 months but not receiving a pneumococcal vaccine. We calculated the proportions of CLSA participants (and 95% CIs) who experienced a MOV of either type. We also used multivariable logistic regression models to identify factors associated with one or the other MOV among each of the risk groups of interest. To adjust for potential confounding in the association between each variable and having experienced a specific MOV (versus no MOV related to the same type of clinical encounter), our models included all sociodemographic factors described above.

Sampling weights for CLSA FU1 data are not available, and baseline weights cannot be applied to these analyses. However, we used age stratification and included sex and province of residence among variables in all our analyses as per CLSA recommendations [25, 26].

All analyses were conducted using the survey data commands in Stata version 17.0 (StataCorp, College Station, TX, USA) [27].

#### Sensitivity analyses

The accuracy of self-reported pneumococcal vaccination status may be lower than that of self-reported influenza vaccination status, given higher potential for poor recall due to the low frequency of administration (once in lifetime versus annually as is the case for influenza vaccination) and the more limited awareness of pneumococcal vaccination. In studies conducted in countries other than Canada, sensitivity (Se) and specificity (Sp) of self-reported pneumococcal vaccination status have been found to vary across population groups (i.e. older adults with or without underlying conditions that increase the risk of IPD and/or adults of variable age with various CMCs), ranging from 73 to 85% and from 83 to 95%, respectively [28–32]. To assess the potential impact of various degrees of misclassification of self-reported pneumococcal vaccination status on estimated associations from Models 1a, 1b, 2a, and 2b, we assumed it to be non-differential with respect to other factors and simulated multiple scenarios based on prespecified values of Se and Sp of self-reported pneumococcal vaccination status. Of note, we examined four scenarios in which sensitivity of self-reporting was lower than specificity (Se 70% and Sp 80%; Se 75% and Sp 85%; Se 80% and Sp 90%; Se 85% and Sp 95%) and four scenarios in which sensitivity of self-reporting was higher than specificity (Se 80% and Sp 70%; Se 85% and Sp 75%; Se 90% and Sp 80%; Se 95% and Sp 85%).

## Results

### Prevalence of pneumococcal vaccination among CLSA participants

Table 1 summarizes the main characteristics of CLSA participants aged ≥65 and those aged 47–64 years with ≥1 CMC, by pneumococcal vaccination status.

**Table 1:** Self-reported pneumococcal vaccination status (vaccinated or unvaccinated during lifetime) among Canadian Longitudinal Study on Aging (CLSA) cohort participants who were considered eligible to receive a pneumococcal vaccine as per Canada’s National Advisory Committee on Immunization (NACI) guidelines, by key sociodemographic characteristics.

Characteristic	Self-reported pneumococcal vaccination in lifetime							
	Individuals aged 65 and older (n = 22,246)				Individuals aged < 65 with at least one CMC (n = 10,815)			
	Vaccinated		Unvaccinated		Vaccinated		Unvaccinated	
	N	% (95% CI)	N	% (95% CI)	N	% (95% CI)	N	% (95% CI)
**Overall**	12,054	54.2 (53.5–54.8)	10,192	45.8 (45.2–46.5)	2,027	18.7 (18.0–19.5)	8,788	81.3 (80.5–82.0)
**Sex at birth**								
Male	5,561	51.1 (50.2–52.1)	5,318	48.9 (47.9–49.8)	933	18.0 (17.0–19.1)	4,250	82.0 (80.9–83.0)
Female	6,488	57.1 (56.2–58.1)	4,865	42.9 (41.9–43.8)	1,093	19.4 (18.4–20.5)	4,536	80.6 (79.5–81.6)
Missing	5	35.7 (15.7–62.4)	9	64.3 (37.6–84.3)	1	33.3 (4.3–84.7)	2	66.7 (15.3–95.7)
**Age group (years)**								
47–55	NA	NA	NA	NA	359	13.2 (12.0–14.5)	2,357	86.8 (85.5–88.0)
55–64	NA	NA	NA	NA	1,668	20.6 (19.7–21.5)	6,431	79.4 (78.5–80.3)
65–74	6,040	47.7 (46.8–48.5)	6,629	52.3 (51.5–53.2)	NA	NA	NA	NA
75–84	4,922	62.3 (61.2–63.3)	2,983	37.7 (36.7–38.8)	NA	NA	NA	NA
≥85	1,092	65.3 (63.0–67.6)	580	34.7 (32.4–37.0)	NA	NA	NA	NA
Missing	0	0	0	0	0	0	0	0
**Race**								
White	11,664	54.4 (53.7–55.1)	9,775	45.6 (44.9–46.3)	1,920	18.9 (18.1–19.7)	8,249	81.1 (80.3–81.9)
Other than white	390	48.3 (44.9–51.8)	417	51.7 (48.2–55.1)	107	16.6 (13.9–19.6)	539	83.4 (80.4–86.1)
Missing	0	0	0	0	0	0	0	0
**Highest education level**								
Less than secondary school education	1,047	55.3 (53.0–57.5)	848	44.7 (42.5–47.0)	92	22.6 (18.2–26.2)	327	78.0 (73.8–81.8)
Secondary school grad., no post-secondary educ.	1,359	53.3 (51.4–55.2)	1,191	46.7 (44.8–48.6)	237	21.2 (18.9–23.7)	881	78.8 (76.3–81.1)
Some post-secondary education	942	53.8 (51.5–56.1)	809	46.2 (43.9–48.5)	172	21.4 (18.7–24.3)	633	78.6 (75.7–81.3)
Post-secondary degree/diploma	8,671	54.3 (53.5–55.0)	7,310	45.7 (45.0–46.5)	1,523	18.0 (17.2–18.8)	6.936	82.0 (81.2–82.8)
Missing	35	50.7 (39.1–62.3)	34	49.3 (37.7–60.9)	3	21.4 (7.1–49.4)	11	78.6 (50.6–92.9)
**Annual household income (in Canadian dollars)**								
Less than $20,000	631	50.2 (47.4–53.0)	626	49.8 (47.0–52.6)	155	30.2 (26.4–34.3)	358	69.8 (65.7–73.6)
$20,000 to <$50,000	3,572	52.6 (51.4–53.8)	3,217	47.4 (46.2–48.6)	359	22.4 (20.4–24.5)	1,246	77.6 (75.5–79.6)
$50,000 to <$100,000	4,542	55.4 (54.3–56.4)	3,660	44.6 (43.6–45.7)	669	19.4 (18.1–20.7)	2,788	80.6 (79.3–81.9)
$100,000 to <$150,000	1,523	55.8 (53.9–57.6)	1,207	44.2 (42.4–46.1)	359	15.1 (13.7–16.6)	2,014	84.9 (83.4–86.3)
$150,000 or higher	731	53.5 (50.8–56.1)	636	46.5 (43.9–49.2)	371	15.7 (14.2–17.2)	1,999	84.3 (82.8–85.8)
Missing	1,055	55.5 (53.3–57.7)	846	44.5 (42.3–46.7)	114	22.9 (19.5–26.8)	383	77.1 (73.2–80.5)
**Marital/partner status**								
Single/Never married/Never lived with a partner	748	50.7 (48.2–53.3)	727	49.3 (46.7–51.8)	279	21.9 (19.7–24.2)	997	78.1 (75.8–80.3)
Married/Common-law	7,446	53.6 (52.8–54.5)	6,433	46.4 (45.5–47.2)	1,394	17.8 (17.0–18.7)	6,435	82.2 (81.3–83.0)
Widowed	2,549	61.5 (60.0–62.9)	1,598	38.5 (37.1–40.0)	89	26.7 (22.2–31.7)	244	73.3 (68.3–77.8)
Divorced/Separated	1.305	47.8 (45.9–49.6)	1,427	52.2 (50.4–54.1)	265	19.3 (17.3–21.5)	1,109	80.7 (78.5–82.7)
Missing	6	46.2 (22.4–71.8)	7	53.8 (28.2–77.6)	0	0	3	100
**Province of residence**								
Newfoundland	472	33.0 (30.6–35.5)	958	67.0 (64.5–69.4)	87	11.3 (9.3–13.8)	681	88.7 (86.2–90.7)
Prince Edward Island	214	43.5 (39.2–47.9)	278	56.5 (52.1–60.8)	37	19.6 (14.5–25.9)	152	80.4 (74.1–85.5)
Nova Scotia	1,014	51.3 (49.1–53.5)	962	48.7 (46.5–50.9)	156	18.6 (16.1–21.4)	681	81.4 (78.6–83.9)
New Brunswick	225	42.0 (37.9–46.2)	311	58.0 (53.8–62.1)	50	18.6 (14.4–23.7)	219	81.4 (76.3–85.6)
Quebec	2,418	58.8 (57.3–60.3)	1,695	41.2 (39.7–42.7)	395	18.9 (17.3–20.6)	1,698	81.1 (79.4–82.7)
Ontario	2,661	54.1 (52.7–55.5)	2,254	45.9 (44.5–47.3)	469	19.4 (17.9–21.1)	1,944	80.6 (78.9–82.1)
Manitoba	1,204	61.8 (59.6–63.9)	745	38.2 (36.1–40.4)	172	18.7 (16.3–21.4)	746	81.3 (78.6–83.7)
Saskatchewan	302	56.3 (52.1–60.5)	234	43.7 (39.5–47.9)	64	25.3 (20.3–31.0)	189	74.7 (69.0–79.7)
Alberta	1,371	62.7 (60.7–64.7)	815	37.3 (35.3–39.3)	249	21.2 (19.0–23.7)	923	78.8 (76.3–81.0)
British Columbia	2,173	52.8 (51.3–54.4)	1,940	47.2 (45.6–48.7)	348	18.3 (16.6–20.1)	1,555	81.7 (79.9–83.4)
Missing	0	0	0	0	0	0	0	0
**Urbanicity of residence**								
Rural	1,579	49.3 (47.5–51.0)	1,627	50.7 (49.0–52.5)	1,723	18.5 (16.7–20.5)	1,339	81.5 (79.5–83.3)
Urban	10,463	55.0 (54.3–55.7)	8,556	45.0 (44.3–45.7)	1,723	18.8 (18.0–19.6)	7,445	81.2 (80.4–82.0)
Missing	12	57.1 (36.0–76.0)	9	42.9 (24.0–64.0)	0	0	4	100

Counts, percentages, and 95% confidence intervals within variable strata are shown for two subgroups of interest: 1) individuals aged 65 and older (n = 22,246), and 2) individuals aged <65 with at least one chronic medical condition (CMC) among those listed in the table (cardiovascular disease, chronic lung disease, cerebrovascular disease, chronic kidney disease, diabetes mellitus, cancer, chronic neurologic condition) (n = 10,815).

Abbreviations: CI, confidence interval; CMC, chronic medical condition; NA, not applicable.

Among 22,246 individuals aged ≥65 years, almost half (10,192, 45.8% [95% CI: 45.2–46.5]) reported not having received pneumococcal vaccine at the time of the survey (2015–2018). We found higher proportions of persons reporting not having received prior pneumococcal vaccination among males versus females, among those aged 65–74 versus older age groups, among those reporting any race other than white versus white participants, among those divorced/separated relative to other marital statuses, among those residing in the Atlantic provinces (New Brunswick, Nova Scotia, Newfoundland, Prince Edward Island) compared to other provinces, and among those residing in rural versus urban areas. About three quarters of those aged ≥65 reported having been diagnosed with ≥1 CMC (16,918, 76.0%). The proportion who had not received pneumococcal vaccine in this group was lower than the proportion among those who did not report having ≥1 CMC (42.9 [95% CI: 42.1–43.6] versus 56.2 [95% CI: 54.8–57.6]). The distribution of CMCs and self-reported pneumococcal vaccination status in this population is provided in S3 Table.

Among participants aged 47–64 who had been diagnosed with ≥1 CMC as previously defined, 81.3% (95% CI: 80.5–82.0; n = 8,788) had not received a pneumococcal vaccine. In this group, proportions unvaccinated were higher among those aged 47–54 versus those aged 55–64, among those with higher income versus lower, and among those living in Newfoundland versus other provinces (Table 1). We observed no meaningful differences in the proportion unvaccinated by sex, race, education, marital status, or urbanicity. Across reported CMCs, proportions unvaccinated ranged from 74.1% (95% CI: 72.6–75.5) for individuals who reported a diagnosis of chronic lung disease to 80.4% (95% CI: 79.4–81.4) for those with cardiovascular disease (S3 Table); the distribution of CMCs by pneumococcal vaccination status is shown in S2 Fig.

### Factors associated with pneumococcal non-vaccination

**Fig 1:**
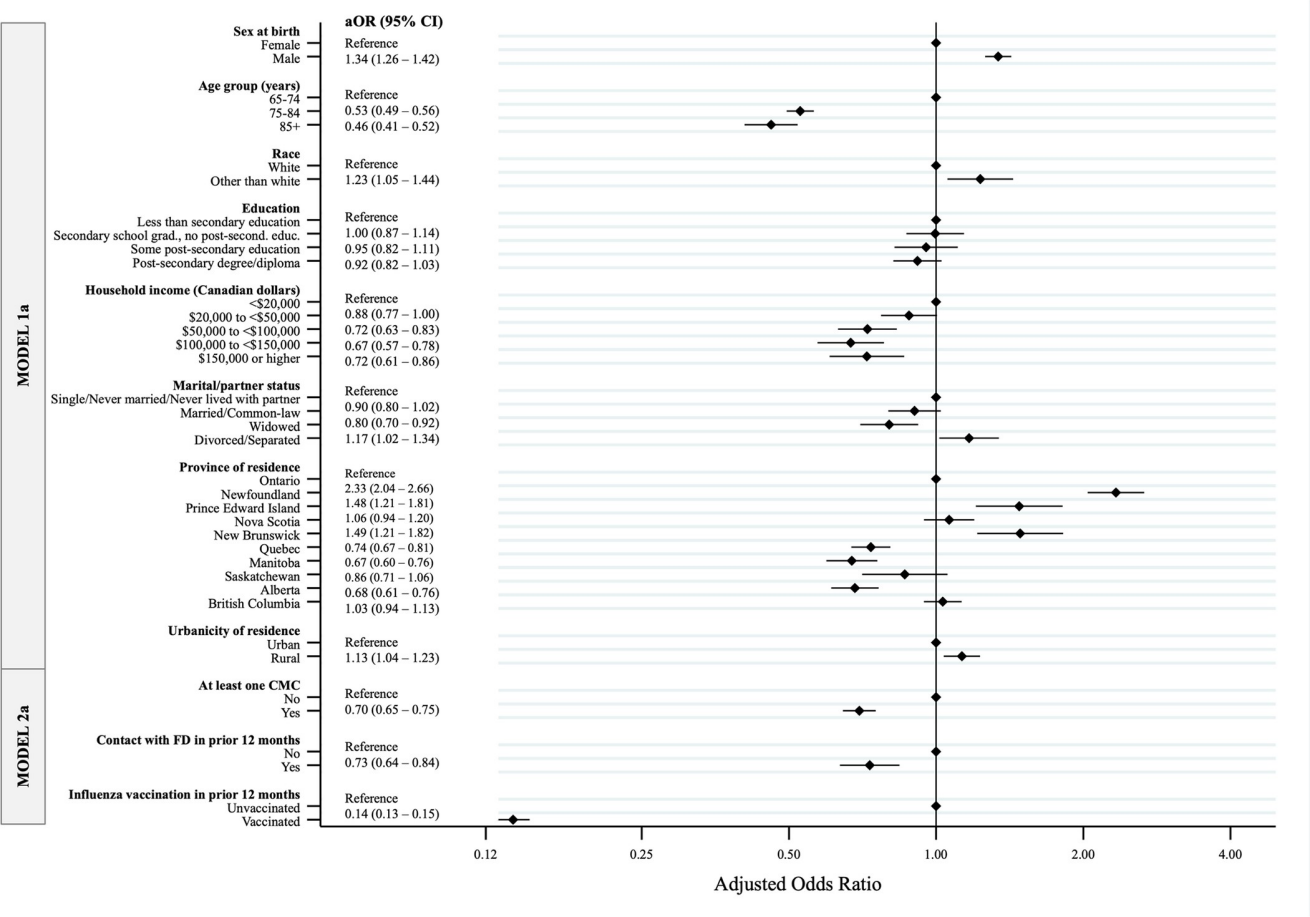
Factors associated with pneumococcal non-vaccination among Canadian Longitudinal Study on Aging (CLSA) participants aged 65 and older who had complete data (n = 19,742). Odds ratios (ORs) and 95% confidence intervals (CIs), plotted on the log-odds ratio scale, were estimated through nested logistic regression models: Model 1 including only sociodemographic characteristics, and Model 2a including all socio-demographics as well as CMC status (having at least one chronic medical condition), recent history of contact with a family doctor and recent influenza vaccination history.

Among individuals aged ≥65, the following sociodemographic factors were independently associated with higher odds of failing to receive a pneumococcal vaccine, after adjusting for all other sociodemographic variables (Model 1a): male sex (aOR = 1.34; 95% CI: 1.26–1.42) versus female, reporting any other race (aOR = 1.23; 95% CI: 1.05–1.44) versus white, being divorced/separated (aOR = 1.17; 95% CI: 1.02–1.34) versus single/never married, and living in rural areas (aOR = 1.13; 95% CI: 1.04–1.23) versus urban (Fig 1). Also, older age groups had lower odds of non-vaccination compared to individuals aged 65–74, with the lowest aOR (0.46; 95% CI: 0.41–0.52) among those aged ≥85 years. Similarly, the odds of non-vaccination were lower for higher income levels versus lowest income, and particularly for those ranging from $100,000 to less than $150,000 (aOR = 0.67; 95% CI: 0.57–0.78). Taking Ontario as reference, participants’ odds of not being vaccinated against pneumococcal disease differed by province of residence, being highest among those residing in Newfoundland (aOR = 2.33; 95% CI: 2.04–2.66), followed by New Brunswick and Prince Edward Island. In contrast, participants residing in Quebec, Manitoba or Alberta had lower odds of non-vaccination than those in Ontario.

According to our fully adjusted model (Model 2a), having received an influenza vaccine in the past year was associated with the lowest odds of being unvaccinated for pneumococcal disease (aOR = 0.14; 95% CI: 0.13–0.15). Those who reported ≥1 CMC or having had contact with a family doctor in the previous 12 months also had lower odds of non-vaccination for pneumococcal disease compared to those who did not (aOR = 0.70 [95% CI: 0.65–0.75] and aOR = 0.73 [95% CI: 0.64–0.84, respectively) (Fig 1).

**Fig 2:**
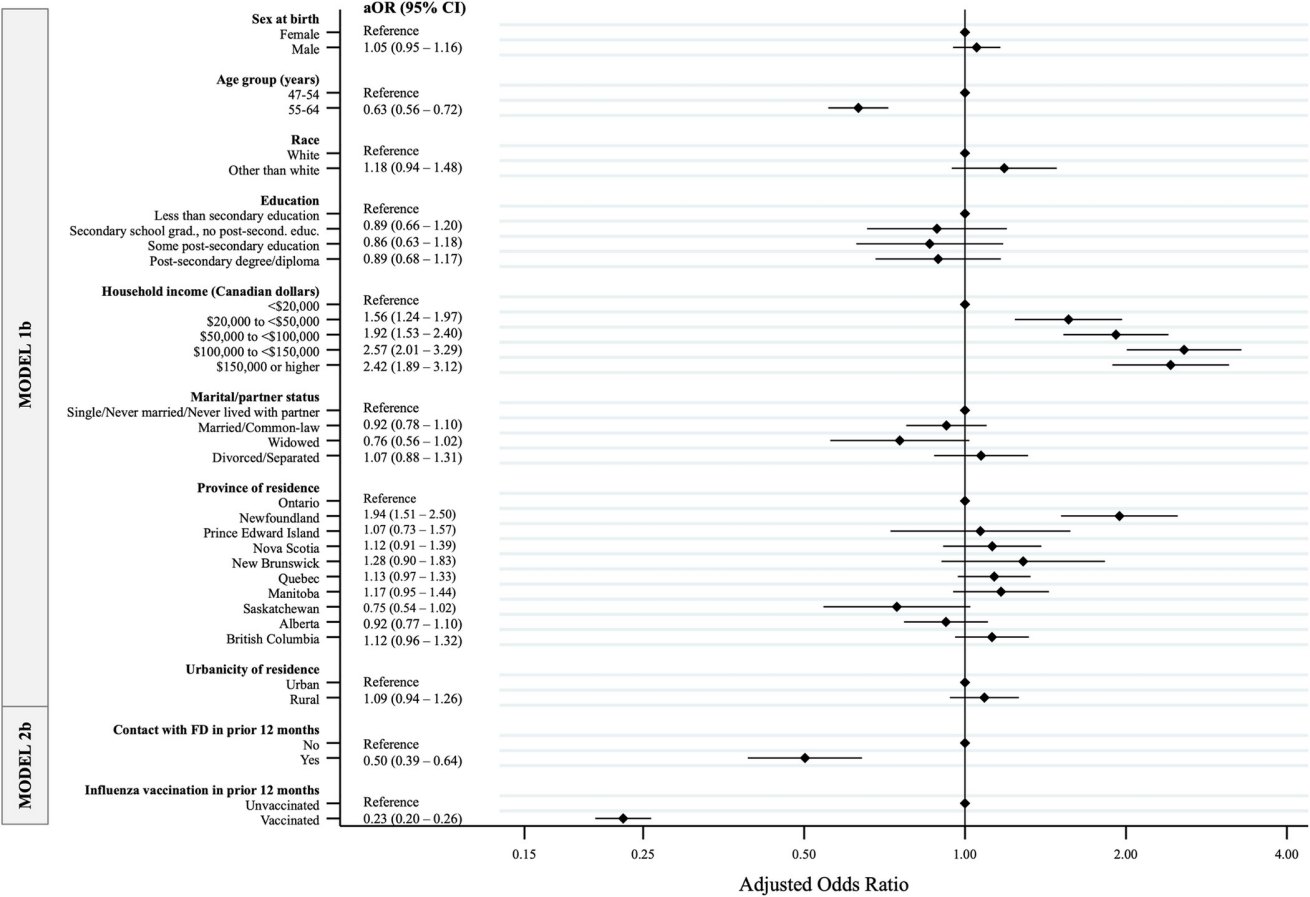
Factors associated with pneumococcal non-vaccination among CLSA participants aged 47–64 who reported having a chronic medical condition (CMC) and had complete data (n = 10,284). Adjusted odds ratios (ORs) and 95% confidence intervals (CIs), plotted on the log-odds ratio scale, were estimated through nested logistic regression models: Model 1 for sociodemographic characteristics; Model 2b for recent history of contact with a family doctor and recent influenza vaccination history, adjusting for socio-demographics.

Regarding factors associated with pneumococcal non-vaccination among individuals aged 47–64 years who had ≥1 CMC (Fig 2), we found that those in higher income groups had higher odds of not being vaccinated, with the strongest association among those with income between $100,000 and less than $150,000 (aOR = 2.57; 95% CI: 2.01–3.29). Residents of Newfoundland were also more likely to be non-vaccinated compared to the reference group (aOR = 1.94; 95% CI: 1.51–2.50), though there were no significant differences observed between other provinces and Ontario. CLSA participants aged 55–64 years with ≥1 CMC were less likely to be non-vaccinated for pneumococcal disease compared to younger ones (aOR = 0.63; 95% CI: 0.56–0.72). Having had contact with a family doctor in the previous year (aOR = 0.50; 95% CI: 0.39–0.64) and receipt of influenza vaccine in the previous year (aOR = 0.23; 95% CI: 0.20–0.26) were both associated with lower odds of pneumococcal non-vaccination (Fig 2).

In the sensitivity analyses, we found that our estimates pertaining to either group (individuals aged ≥65 or those aged 47–64 who had ≥1 CMC) were reasonably robust to various degrees of misclassification of the outcome that might result from poor recall (S4–S7 Tables). However, in situations with the lowest specificity of self-reported pneumococcal vaccination status, the estimated associations were closer to the null compared to those obtained from primary analyses.

### Missed opportunities for pneumococcal vaccination

#### MOV related to receipt of influenza vaccination

**Fig 3:**
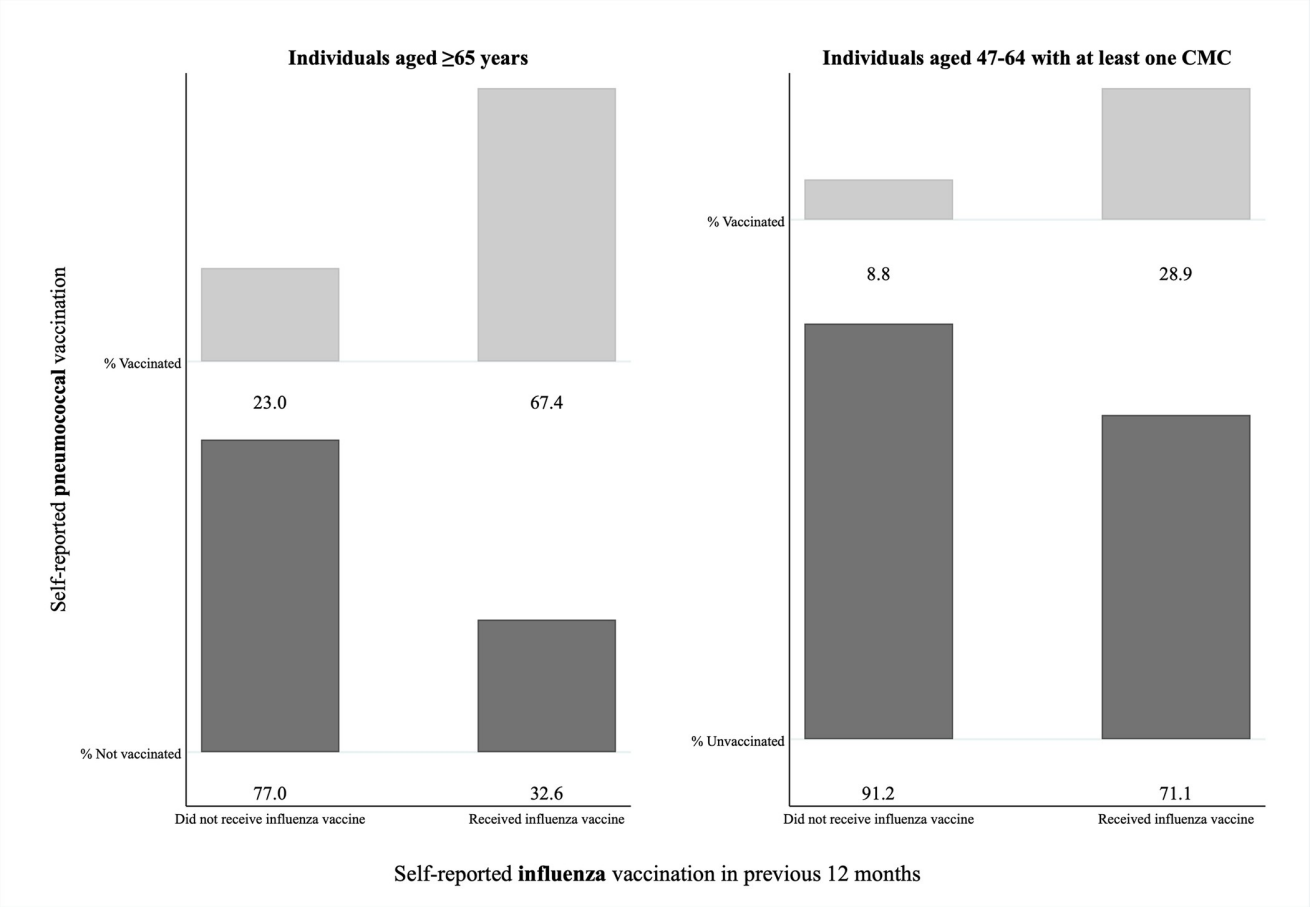
Self-reported pneumococcal vaccination status among CLSA participants aged ≥65 years (n = 22,216) and among those aged 47–64 with at least one chronic medical condition (CMC) (n = 10,811), stratified by receipt of influenza vaccination in the previous 12 months. Percentages of individuals who were vaccinated or non-vaccinated against pneumococcal disease are shown for each category. For each of the two groups eligible for pneumococcal vaccination, those with no available data concerning self-reported influenza vaccination were excluded (i.e. 30 and 4, respectively).

Fig 3 graphically shows the proportion of pneumococcal vaccinated and non-vaccinated individuals among those who had received an influenza vaccine in the previous 12 months versus those who did not.

We estimated that, among participants aged ≥65 years who reported receipt of influenza vaccination in the 12 months prior, males (aOR = 1.41 [95% CI: 1.30–1.52] relative to females) and those residing in any of the Atlantic provinces (aOR = 2.26 [95% CI: 1.94–2.64] for Newfoundland versus Ontario) were more likely to experience a missed opportunity for pneumococcal vaccination (Table 2). Instead, older age groups (versus those aged 65–74), widowed individuals, and those outside the Atlantic provinces had lower odds of missing that opportunity.

**Table 2:** Factors associated with missed opportunity for pneumococcal vaccination among CLSA participants aged ≥65 years and those aged 47–64 with at least one of select chronic medical conditions (CMC)* who reported receiving an influenza vaccine in the previous 12 months.

Variable	Reported receiving an influenza vaccine in the previous 12 months	
	Individuals aged ≥65 years (n = 14,267)	Individuals aged 47–64 with at least one CMC* (n = 5,096)
	*aOR (95% CI)*	*aOR (95% CI)*
**Sex at birth**		
Female	Reference	Reference
Male	1.41 (1.30–1.52)	1.01 (0.89–1.15)
**Age group (years)**		
47–55	NA	Reference
55–64	NA	0.71 (0.61–0.84)
65–74	Reference	NA
75–84	0.60 (0.55–0.65)	NA
≥85	0.57 (0.49–0.66)	NA
**Race**		
White	Reference	Reference
Other than white	1.11 (0.90–1.35)	1.11 (0.85–1.47)
**Highest education level**		
Less than secondary school education	Reference	Reference
Secondary school grad., no post-secondary educ.	0.94 (0.79–1.13)	0.73 (0.50–1.09)
Some post-secondary education	0.98 (0.81–1.19)	0.76 (0.50–1.14)
Post-secondary degree/diploma	0.94 (0.81–1.09)	0.84 (0.59–1.19)
**Annual household income (in Canadian dollars)**		
Less than $20,000	Reference	Reference
$20,000 to <$50,000	0.94 (0.78–1.13)	1.46 (1.07–1.98)
$50,000 to <$100,000	0.86 (0.71–1.04)	2.00 (1.48–2.69)
$100,000 to <$150,000	0.86 (0.70–1.06)	2.62 (1.90–3.62)
$150,000 or higher	0.94 (0.74–1.19)	2.58 (1.86–3.57)
**Marital/partner status**		
Single/Never married/Never lived with a partner	Reference	Reference
Married/Common-law	0.92 (0.78–1.09)	1.03 (0.83–1.28)
Widowed	0.82 (0.68–0.98)	0.92 (0.63–1.35)
Divorced/Separated	1.09 (0.90–1.30)	1.09 (0.84–1.40)
**Province of residence**		
Ontario	Reference	Reference
Newfoundland	2.26 (1.94–2.64)	2.01 (1.48–2.73)
Prince Edward Island	1.71 (1.36–2.16)	1.46 (0.90–2.36)
Nova Scotia	1.20 (1.06–1.36)	1.55 (1.21–1.98)
New Brunswick	1.38 (1.09–1.75)	1.24 (0.82–1.88)
Quebec	0.36 (0.32–0.41)	0.77 (0.63–0.94)
Manitoba	0.55 (0.48–0.64)	1.25 (0.98–1.61)
Saskatchewan	0.56 (0.43–0.73)	0.61 (0.41–0.90)
Alberta	0.50 (0.43–0.58)	1.06 (0.86–1.32)
British Columbia	0.84 (0.76–0.94)	1.10 (0.91–1.33)
**Urbanicity of residence**		
Urban	Reference	Reference
Rural	1.04 (0.93–1.16)	1.00 (0.83–1.21)

Adjusted odds ratios (aORs) and 95% confidence intervals (CIs) were estimated from logistic regression models including all sociodemographic variables, restricting to individuals with complete data.

Abbreviations: aOR, adjusted odds ratio; CI, confidence interval; CMC, chronic medical condition; NA, not applicable.

*The following chronic medical conditions were considered: cardiovascular disease (including prior heart attack/myocardial infarction, angina or chest pain due to heart disease, hypertension), chronic lung disease (including emphysema, chronic bronchitis, chronic obstructive pulmonary disease, chronic changes in lungs due to smoking, asthma), cerebrovascular disease (including stroke and transient ischemic attack), chronic kidney disease or failure, diabetes mellitus, cancer, and chronic neurologic condition (including dementia, Alzheimer’s disease, Parkinson’s disease, multiple sclerosis).

When we examined individuals aged 47–64 with ≥1 CMC, we found that having higher income (versus lowest) and residing in Newfoundland or Nova Scotia (compared to the reference group) were associated with higher odds of not getting a pneumococcal vaccine despite having received influenza vaccination in the previous 12 months (Table 2). Only those in Quebec or Saskatchewan (relative to Ontario residents) and those in the 55–64 age range (versus younger ages) were less likely to miss an opportunity for pneumococcal vaccination.

#### Missed opportunity related to prior contact with a family doctor

**Fig 4:**
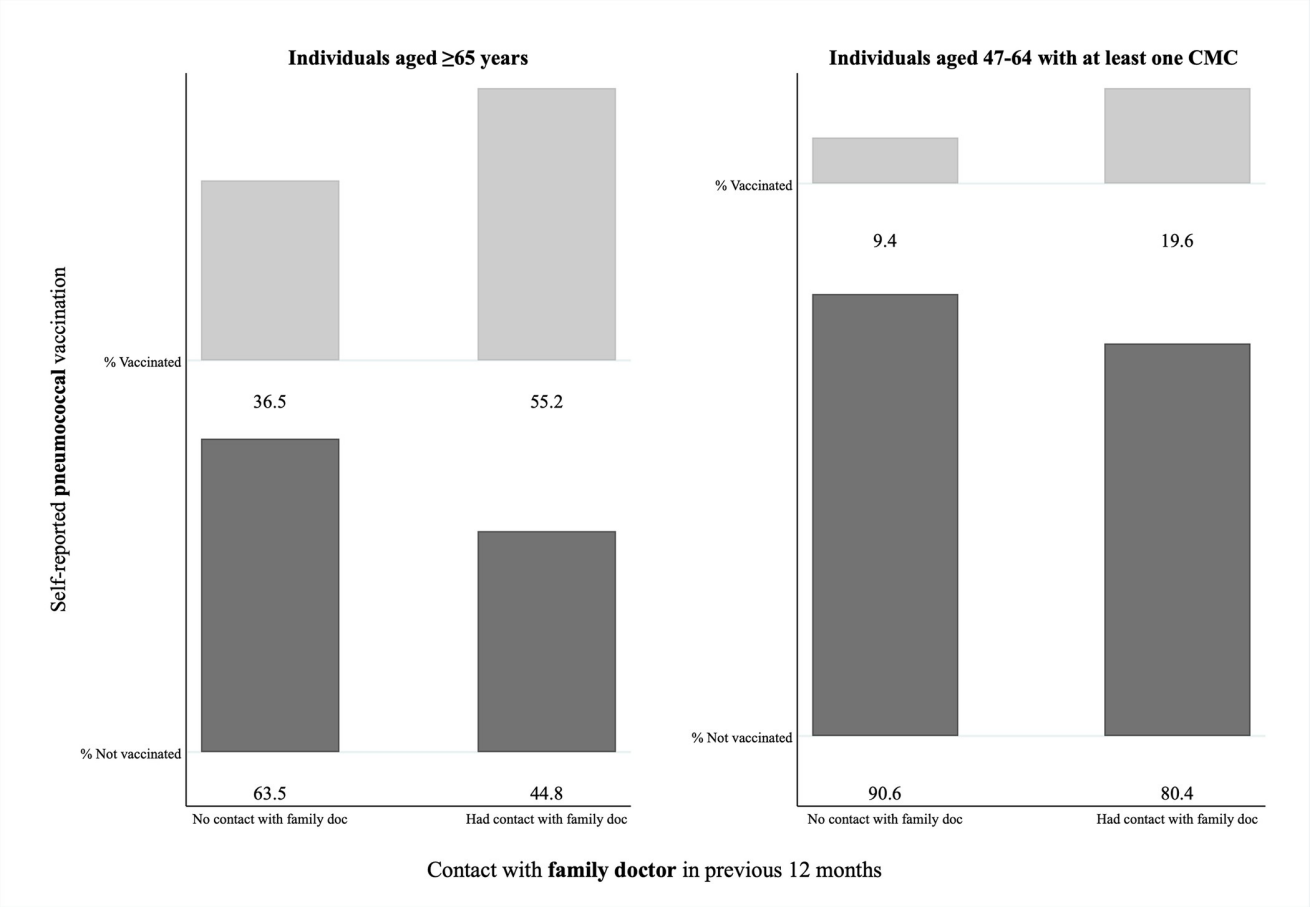
Self-reported pneumococcal vaccination status among CLSA participants aged ≥65 years (n = 22,234) and among those aged 47–64 with at least one chronic medical condition (CMC) (n = 10,807), stratified by history of contact with a family doctor in the previous 12 months. Percentages of individuals who were vaccinated or non-vaccinated against pneumococcal disease are shown for each category. For each of the two groups eligible for pneumococcal vaccination, those with no available data concerning prior contact with a family doctor were excluded (i.e., 12 and 8, respectively).

Proportions of participants who received or did not receive a pneumococcal vaccination among those who reported having had contact with a family physician in the preceding 12 months are presented in Fig 4.

We estimated that, among individuals aged ≥65 who had had contact with a family doctor, participants were more likely to experience a MOV if they were male (aOR = 1.33; 95% CI: 1.25–1.42), of a racial group other than white (aOR = 1.22; 95% CI: 1.04–1.42), divorced/separated (aOR = 1.18; 95% CI: 1.02–1.37), resident in rural areas (aOR = 1.14; 95% CI: 1.04–1.24), or resident in the Atlantic provinces except for Nova Scotia, with the highest odds for those living in Newfoundland (aOR = 2.36; 95% CI: 2.06–2.69). Conversely, being 75–84 or ≥85 years, widowed, from higher income levels, or resident outside the Atlantic provinces except for British Columbia were all associated with lower odds of missing an opportunity for vaccination when visiting a family physician (Table 3).

**Table 3:** Factors associated with missed opportunity for pneumococcal vaccination among CLSA participants aged ≥65 years and those aged 47–64 with at least one of select chronic medical conditions (CMC)* who reported having had contact with a family doctor in the previous 12 months.

Variable	Reported having had contact with a family doctor in the previous 12 months
	Individuals aged ≥65 years (n = 19,117)	Individuals aged 47–64 with at least one CMC* (n = 9,446)
	*aOR (95% CI)*	*aOR (95% CI)*
**Sex at birth**		
Female	Reference	Reference
Male	1.33 (1.25–1.42)	1.03 (0.93–1.15)
**Age group (years)**		
47–55	NA	Reference
55–64	NA	0.65 (0.57–0.75)
65–74	Reference	NA
75–84	0.55 (0.52–0.59)	NA
≥85	0.49 (0.43–0.56)	NA
**Race**		
White	Reference	Reference
Other than white	1.22 (1.04–1.42)	1.20 (0.95–1.51)
**Highest education level**		
Less than secondary school education	Reference	Reference
Secondary school grad., no post-secondary educ.	0.95 (0.83–1.10)	0.93 (0.68–1.27)
Some post-secondary education	0.94 (0.80–1.09)	0.88 (0.63–1.21)
Post-secondary degree/diploma	0.90 (0.80–1.00)	0.89 (0.67–1.17)
**Annual household income (in Canadian dollars)**		
Less than $20,000	Reference	Reference
$20,000 to <$50,000	0.90 (0.79–1.03)	1.60 (1.26–2.03)
$50,000 to <$100,000	0.75 (0.65–0.86)	1.94 (1.53–2.45)
$100,000 to <$150,000	0.70 (0.60–0.82)	2.58 (2.00–3.33)
$150,000 or higher	0.75 (0.63–0.90)	2.45 (1.90–3.18)
**Marital/partner status**		
Single/Never married/Never lived with a partner	Reference	Reference
Married/Common-law	0.92 (0.81–1.05)	0.95 (0.80–1.14)
Widowed	0.81 (0.70–0.93)	0.75 (0.55–1.02)
Divorced/Separated	1.18 (1.02–1.37)	1.08 (0.88–1.33)
**Province of residence**		
Ontario	Reference	Reference
Newfoundland	2.36 (2.06–2.49)	2.07 (1.59–2.67)
Prince Edward Island	1.38 (1.12–1.70)	1.08 (0.72–1.61)
Nova Scotia	1.06 (0.95–1.20)	1.15 (0.93–1.42)
New Brunswick	1.50 (1.23–1.85)	1.34 (0.93–1.92)
Quebec	0.72 (0.66–0.80)	1.14 (0.97–1.34)
Manitoba	0.68 (0.61–0.77)	1.19 (0.97–1.47)
Saskatchewan	0.84 (0.68–1.02)	0.82 (0.59–1.15)
Alberta	0.68 (0.61–0.77)	0.93 (0.77–1.11)
British Columbia	1.03 (0.94–1.13)	1.14 (0.97–1.35)
**Urbanicity of residence**		
Urban	Reference	Reference
Rural	1.14 (1.04–1.24)	1.08 (0.92–1.25)

Adjusted odds ratios (aORs) and 95% confidence intervals (CIs) were estimated from logistic regression models including all sociodemographic variables, restricting to individuals with complete data.

Abbreviations: aOR, adjusted odds ratio; CI, confidence interval; CMC, chronic medical condition; NA, not applicable.

*The following chronic medical conditions were considered: cardiovascular disease (including prior heart attack/myocardial infarction, angina or chest pain due to heart disease, hypertension), chronic lung disease (including emphysema, chronic bronchitis, chronic obstructive pulmonary disease, chronic changes in lungs due to smoking, asthma), cerebrovascular disease (including stroke and transient ischemic attack), chronic kidney disease or failure, diabetes mellitus, cancer, and chronic neurologic condition (including dementia, Alzheimer’s disease, Parkinson’s disease, multiple sclerosis).

Focusing on participants aged 47–64 who had ≥1 CMC, higher household income levels (versus less than $20,000) and being resident in Newfoundland (versus Ontario) were associated with higher odds of not getting a pneumococcal vaccine among those who had had contact with a family physician in the previous 12 months, whereas those aged 55–64 were less likely to be non-vaccinated than younger individuals (Table 3).

## Discussion

Despite the modest increase in coverage over the past decade, pneumococcal vaccine uptake among Canadian adults who are at higher risk of IPD remains low. We analysed cross-sectional data collected in 2015–2018 from a large cohort of Canadian residents to better understand pneumococcal vaccination uptake, factors associated with non-vaccination, and MOV among those aged ≥65 years and among those aged 47–64 with ≥1 CMCs. We found that 45.8% of adults aged ≥65 and 81.3% of those aged 47–64 with ≥1 CMC reported never having received a pneumococcal vaccine in their lifetime, though the proportions who remained non-vaccinated were lower among older age groups in both subpopulations. Reporting receipt of influenza vaccination in the previous 12 months and–to a much lesser extent–reporting having had contact with a family doctor over the same period were the strongest predictors of pneumococcal vaccination in both population groups. Several sociodemographic factors were also associated with non-vaccination among older adults. Furthermore, we found a high degree of heterogeneity in pneumococcal vaccine uptake across provinces, with participants aged ≥65 from the Atlantic provinces having higher odds of being unvaccinated. Among individuals aged 47–65 who had ≥1 CMC, the only sociodemographic factors associated with pneumococcal non-vaccination were having a higher income (versus less than $20,000) and residing in Newfoundland (versus Ontario). These differences across geographic areas likely reflect the diversity of challenges and approaches to vaccine deployment observed across the country. While almost all provinces have directives to facilitate the administration of PPV23 in long-term care facilities [17], the settings where the pneumococcal vaccine is administered to community-dwelling seniors and other adults at risk (e.g. doctors’ offices, public health sites, pharmacies) vary by province. For example, some providers (e.g. pharmacies), might offer the service at a cost, making accessibility financially heterogenous across Canada [17]. Additionally, little is known about awareness of pneumococcal vaccination eligibility and vaccination willingness among older Canadian adults, but these factors may also be a concern [33, 34].

While some sociodemographic characteristics beyond participants’ province of residence were associated with receipt of pneumococcal vaccine among older adults, the magnitude of most of these associations is modest, suggesting that low uptake of pneumococcal vaccination is a cross-cutting issue among the risk groups we investigated. Moreover, the very low prevalence of self-reported pneumococcal vaccine uptake among adults aged 47–64 who had ≥1 CMC is particularly striking and might in part result from lack of awareness. At least a subset of these adults with CMCs are followed by specialists, including respirologists, which represents an opportunity to counsel if not provide the vaccine. A 2020–2021 survey conducted by the Public Health Agency of Canada has shown that “never having heard of this vaccine” is among the top three reasons for pneumococcal non-vaccination [15].

Our analyses also indicate that a substantial proportion of individuals who would benefit from pneumococcal vaccination according to recommendations missed an opportunity to get vaccinated. For those individuals who are eligible for both influenza and pneumococcal vaccines and who received the former but not the latter, efforts could be deployed to ensure that both vaccines are recommended and administered on the same occasion. We found that 32.6% of those aged ≥65 and 71.1% of those aged 47–64 with ≥1 CMC missed out on receiving pneumococcal vaccine even though they recently received influenza vaccine. We also found that 44.8% of those aged ≥65 and 80.4% of those aged 47–64 with ≥1 CMC missed out despite recent contact with a family doctor. The issue of missed pneumococcal vaccination opportunities in Canada has been explored previously, most notably through some small-sample studies conducted in the province of Alberta [18, 35, 36]. Our study is the first to provide evidence on the extent of the problem and its associated factors nationwide. Lack of pneumococcal vaccination among those who reported receiving an influenza vaccine might be due to issues with availability (as could be the case of some pharmacies that may not offer the vaccine), awareness, acceptance, or a combination of these factors. With respect to those who reported contact with a family doctor, reasons for missing the opportunity to receive a pneumococcal vaccine even though eligible could be related to the fact that many individuals typically seek care for acute ailments (e.g. upper respiratory tract infections) that may contraindicate the immediate administration of any vaccine at that visit. Moreover, previous research has shown that knowledge of and ability to deliver key vaccinations likely varies across providers and settings and may thus contribute to non-vaccination among high-risk populations [37, 38]. It is also worth highlighting that roughly 15% of Canadians do not have a regular healthcare provider and can only count on walk-in clinics that, however, do not offer the same degree of continuity of care that might improve the provider-patient relationship and favor the implementation of preventative actions such as immunizations [39, 40]. Examining MOV requires further investigation to better understand the reasons why vaccination was not offered and/or administered to someone eligible, which could suggest opportunities for designing and testing interventions at the provider, patient, community, or healthcare system level to increase uptake. Leveraging influenza vaccination sites to counsel and/or provide pneumococcal vaccination, remunerating family doctors for counselling, adding pneumococcal vaccination as a task in electronic health records, or sending reminders such as those for cancer screening could be practical policy recommendations to increase uptake [41–44].

Previous studies conducted in other countries most often reported the average number of MOV per person over a specified time without examining in detail individual-level features that were associated with that. Additionally, the definition of MOV is subject to a lot of variation by setting and research context, primarily as a function of type of available data and healthcare system structure and mechanisms. For instance, in a US study using the 2015 National Health Interview Survey data, an average of 5.2 MOV–defined as any healthcare encounter–were reported by participants aged ≥65 years who had never received a pneumococcal vaccine [45]. Another recent study from Australia found that missed opportunities for both influenza and pneumococcal vaccination in adults aged ≥45 often occurred, but only hospital-based consultations were considered as potential opportunities to promote and deliver vaccines [46]. Suboptimal pneumococcal vaccination rates and high frequency of MOV have also been documented across European countries [47].

The main strength of our study is that it fills important knowledge gaps by examining multiple factors associated with pneumococcal non-vaccination and MOV among seniors as well as middle-aged adults with ≥1 CMC in Canada. Our assessment was based on a very large cohort of Canadian residents [21–23], which has been instrumental in generating key evidence on multiple health aspects related to aging and has provided us with an invaluable platform to address highly relevant questions pertaining to adult vaccinations including pneumococcal vaccines and others [48, 49]. Furthermore, the proportion of missing data on prior receipt of pneumococcal vaccination among CLSA participants was very low (only 5% of those aged ≥65 and none of those aged 47–64 with ≥1 CMC).

This study also has some limitations. Despite being a rigorously designed national study, CLSA cohort recruitment is known to have resulted in a sample with higher socio-economic status and education, and less frailty and dementia, than the general older adult population in Canada, which may impact generalizability [50]. Also, primary data collection related to our analysis was based on participants’ self-reporting, thus potentially resulting in misclassification due to factors such as recall bias. This is especially concerning for pneumococcal vaccination status, whose accuracy may not be optimal as this vaccine usually requires a single dose with no subsequent boosters [28–32]. It is reassuring that our uptake estimates aligned with national statistics, and our regression models were robust to multiple sensitivity analyses, suggesting that potential outcome misclassification would be unlikely to have anything more than a minor impact on our results. Nonetheless, while a very small proportion of CLSA participants had missing information on self-reported pneumococcal vaccination status, it is possible that some reported their vaccination status incorrectly due to lack of knowledge about this vaccine or other factors. Future follow-up surveys should consider the inclusion of questions aimed at investigating participants’ knowledge about and familiarity with pneumococcal vaccines. Second, our list of CMCs grouped into eight categories included only a subset of those conditions for which pneumococcal vaccination is recommended in Canada [8, 9]. We could not include all possible CMCs as not all are reported in the CLSA. Yet, the conditions considered in our study represent the major CMCs affecting middle-aged and older adults, such as diabetes, hypertension, or chronic obstructive pulmonary disease. Third, the extent of MOV could have been underestimated as we only examined contacts with a family doctor and receipt of influenza vaccine in the previous year. It is possible that participants had had other relevant healthcare encounters (e.g. specialist visits) during or before that time, which still did not result in getting a pneumococcal vaccine. Relatedly, our data did not allow us to explore why participants had missed an opportunity to receive a pneumococcal vaccine, which could be due to provider, participant, health system factors or a combination of these related to awareness of vaccine eligibility, availability of the vaccine, and vaccination willingness. Reasons why opportunities for pneumococcal vaccination are missed during clinical encounters is a key area for future research so that interventions can be designed and tested to address the main barriers.

## Conclusions

Pneumococcal vaccination coverage among adults at increased risk of IPD in Canada remains at sub-optimal levels and the ongoing coronavirus disease 2019 (COVID-19) pandemic, which has strained the healthcare system and severely impacted older adults, increases the urgency of preventing other diseases that lead to hospitalization and death. Understanding the reasons for pneumococcal non-vaccination and developing effective ways to reduce MOV so that eligible individuals can be vaccinated is crucial to increasing pneumococcal vaccine uptake among those at risk and tackling one of the top ten causes of death among adults.

## Supporting information

S1 Fig Canadian Longitudinal Study on Aging (CLSA) participants’ flow from the baseline CLSA study through to follow-up 1 and inclusion into our analyses as relevant. (PDF)

S2 Fig Distribution of chronic medical conditions (CMCs) among 10,815 Canadian Longitudinal Study on Aging (CLSA) participants aged 47–64 who had at least one CMC, by self-reported pneumococcal vaccination status. (PDF)

S1 Table Characteristics of pneumococcal vaccination programs across Canadian provinces. (PDF)

S2 Table Description of survey questions, response options, and study variables. (PDF)

S3 Table Distribution of chronic medical conditions (CMC) among individuals eligible for pneumococcal vaccination, by self-reported pneumococcal vaccination status (vaccinated or unvaccinated during lifetime). (PDF)

S4 Table Results of sensitivity analyses of factors associated with pneumococcal non-vaccination among 19,742 Canadian Longitudinal Study on Aging (CLSA) participants aged 65 years and older who had complete data on all variables of interest–Scenarios 1–4. (PDF)

S5 Table Results of sensitivity analyses of factors associated with pneumococcal non-vaccination among 19,742 Canadian Longitudinal Study on Aging (CLSA) participants aged 65 years and older who had complete data on all variables of interest–Scenarios 5–8. (PDF)

S6 Table Results of sensitivity analyses of factors associated with pneumococcal non-vaccination among 10,284 Canadian Longitudinal Study on Aging (CLSA) participants aged under 65 years who had been diagnosed with at least one chronic medical condition that made them eligible for pneumococcal vaccination–Scenarios 1–4. (PDF)

S7 Table Results of sensitivity analyses of factors associated with pneumococcal non-vaccination among 10,284 Canadian Longitudinal Study on Aging (CLSA) participants aged under 65 years who had been diagnosed with at least one chronic medical condition that made them eligible for pneumococcal vaccination–Scenarios 5–8. (PDF)
