# Supplementary material for: Pneumococcal vaccination uptake and missed opportunities for vaccination among Canadian adults: A cross-sectional analysis of the Canadian Longitudinal Study on Aging (CLSA)
Source: PLoS One. 2022 Oct 14;17(10):e0275923. doi: 10.1371/journal.pone.0275923 (PMC9565727; doi:10.1371/journal.pone.0275923)

S1 Fig: **Canadian Longitudinal Study on Aging (CLSA) participants' flow from the baseline CLSA study through to follow-up 1 and inclusion into our analyses as relevant.**

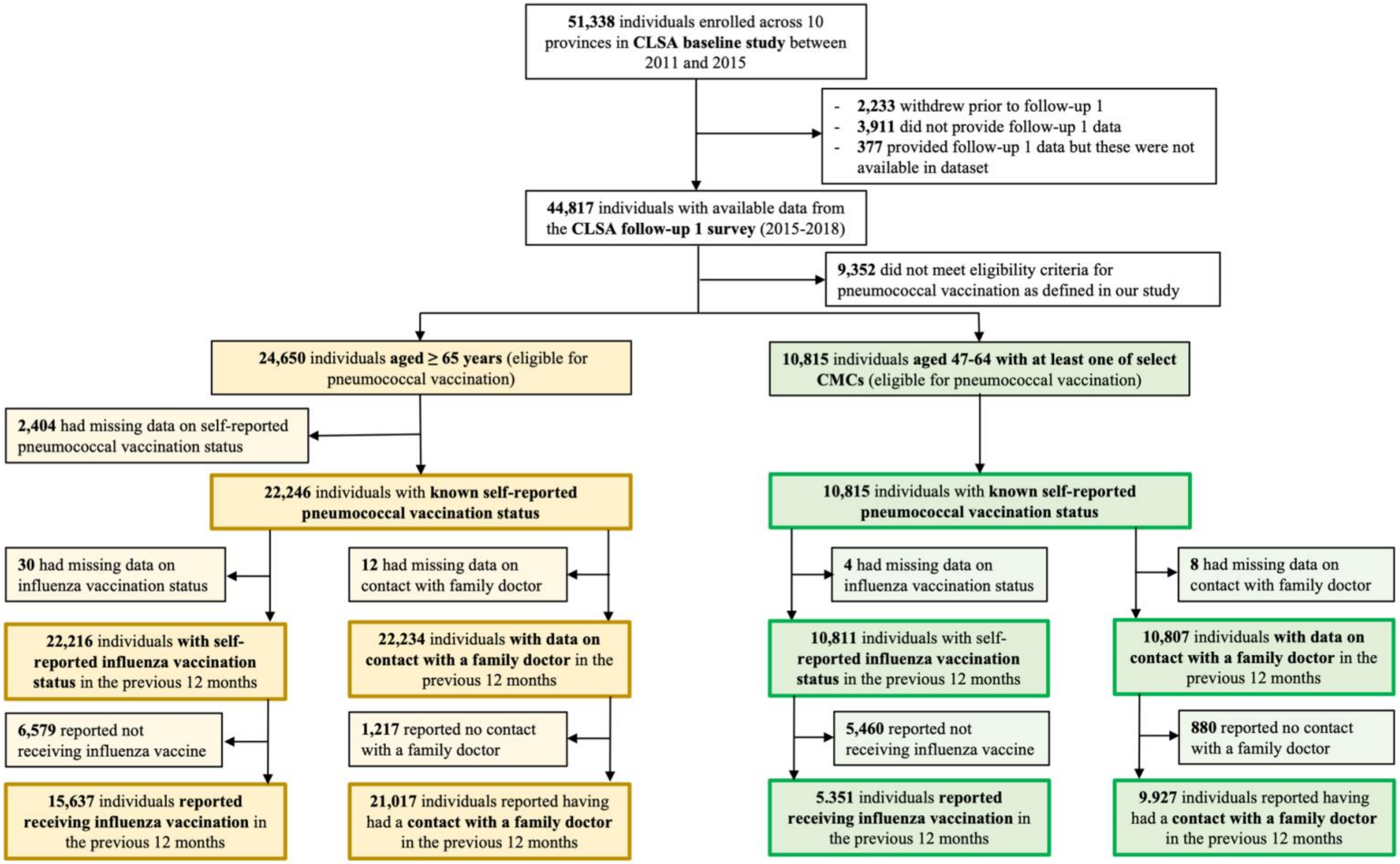

Supplement: S1 Fig — (PDF) [file pone.0275923.s001.pdf]
