# Supplementary material for: Pneumococcal vaccination uptake and missed opportunities for vaccination among Canadian adults: A cross-sectional analysis of the Canadian Longitudinal Study on Aging (CLSA)
Source: PLoS One. 2022 Oct 14;17(10):e0275923. doi: 10.1371/journal.pone.0275923 (PMC9565727; doi:10.1371/journal.pone.0275923)

S2 Fig: **Distribution of chronic medical conditions (CMCs) among 10,815 Canadian Longitudinal Study on Aging (CLSA) participants aged 47-64 who had at least one CMC, by self-reported pneumococcal vaccination status.**

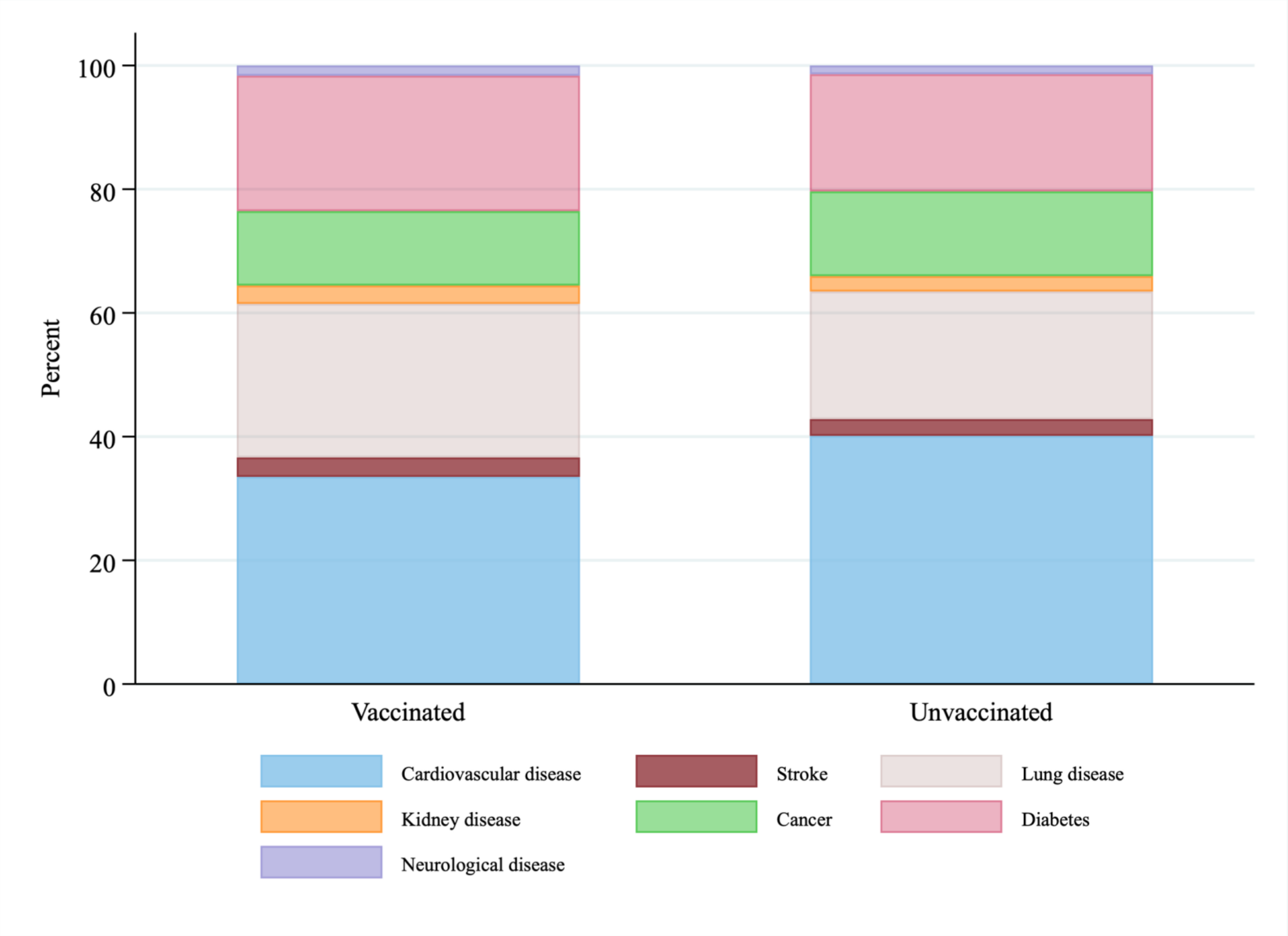

Supplement: S2 Fig — (PDF) [file pone.0275923.s002.pdf]
