# Supplementary material for: Pneumococcal vaccination uptake and missed opportunities for vaccination among Canadian adults: A cross-sectional analysis of the Canadian Longitudinal Study on Aging (CLSA)
Source: PLoS One. 2022 Oct 14;17(10):e0275923. doi: 10.1371/journal.pone.0275923 (PMC9565727; doi:10.1371/journal.pone.0275923)
Supplement: S1 Table — (PDF) [file pone.0275923.s003.pdf]

S1 Table: Characteristics of adult pneumococcal vaccination programs across Canadian provinces. For all eligible categories among individuals aged 65 and older and among those aged 18-64 who are at increased risk of invasive pneumococcal disease (IPD), we provide the start year of the program, whether pneumococcal vaccination is covered by public funding and whether the vaccine is available in pharmacies.

**Province [References]**	**Pneumococcal vaccination for people aged 65 and older**		**Pneumococcal vaccination for people aged 18-64 who are at increased risk of IPD**			**Availability in pharmacies[#]**
	**Program start year**	**Public funding**	**Groups**	**Program start year[*]**	**Public funding**	
Newfoundland [1, 2]	1999	Yes	People with chronic conditions requiring regular medical treatment and follow-up, such as:<br>- Chronic cardiac disease<br>- Chronic respiratory disease<br>- Cirrhosis<br>- Asplenia or splenic dysfunction<br>- Sickle-cell disease<br>- Nephrotic syndrome<br>- Immunosuppression (e.g. induced through HIV infection and other conditions)<br>- Diabetes mellitus<br>- Alcoholism.	1980	Yes	No
			Residents of long-term care or residential facilities			
			Indigenous populations			
			People receiving or with cochlear implants			
Prince Edward Island [3]	Not retrieved	Yes	Immunocompetent people with one or more of the following conditions (high risk):<br>- Asthma that required treatment in the preceding 12 months<br>- CSF leak<br>- Neurological conditions that may impair clearance of oral secretions<br>- Chronic cardiac disease<br>- Chronic pulmonary disease<br>- Diabetes mellitus<br>- Alcoholism<br>- Illicit drug use<br>- Tobacco smoking	Not retrieved	Yes	Yes
			Immunocompromised people with one or more of the following conditions (highest risk):<br>- Chronic liver disease (including hepatic cirrhosis due to any cause)<br>- Chronic kidney disease/dialysis<br>- Sickle-cell disease or other hemoglobinopathies<br>- Congenital immunodeficiency involving any part of the immune system<br>- Asplenia (functional or anatomic)<br>- Immunocompromising therapy including use of long-term corticosteroids (other than by inhalation, topical, or injection into a joint), chemotherapy, radiation therapy, post-organ transplant therapy, and certain anti-rheumatic drugs<br>- HIV infection<br>- HSCT<br>- Malignant neoplasms including leukemia and lymphoma<br>- Nephrotic syndrome<br>- Solid organ transplant (candidate or recipient)			

Province [References]	Pneumococcal vaccination for people aged 65 and older		Pneumococcal vaccination for people aged 18-64 who are at increased risk of IPD			Availability in pharmacies#
	Program start year	Public funding	Groups	Program start year*	Public funding	
Nova Scotia [4-6]	1997	Yes	Immunocompromised people due to one or more of the following reasons (highest risk:<br>- Cancer<br>- Congenital immunodeficiency<br>- HIV infection<br>- Impaired spleen function<br>- HSCT<br>- Solid organ transplant<br>- Immunosuppressive therapy<br>Immunocompetent people with one or more of the following conditions (high risk):<br>- Chronic CSF leak<br>- Liver disease<br>- Pulmonary disease, excluding asthma<br>- Neurological conditions<br>- Renal disease<br>- Cochlear implant<br>- Diabetes mellitus<br>- Heart disease<br>- Cystic fibrosis<br>- Alcoholism or illicit drug use<br>- Homelessness	2015	Yes	Yes
			Residents of long-term care facilities			
New Brunswick [7]	1984	Yes	Immunocompromised individuals	2014	Yes	Yes
			People with CMCs that increase the risk of IPD			
			Homelessness			
			Illicit drug use or alcoholism			
			Residents of long-term care facilities			
Quebec [8, 9]	2000	Yes	People with impaired spleen function	1999	Yes	Yes
			People receiving cochlear implant surgery			
			People with chronic diseases such as diabetes, cancer, heart/respiratory/kidney disease			
			People aged 50 and older who have asthma requiring regular medical follow-up			
			Immunocompromised people			
			People with a medical condition causing difficulties in the evacuation of respiratory secretions			
			Homelessness			
			Regular use of illicit drugs with a deteriorated health condition or precarious living conditions			
Ontario [10-12]	1996	Yes (at healthcare facilities)	People with impaired spleen function	1996	Yes	Yes
			People with chronic cardiac disease			
			People with chronic CSF leak			
			Candidates and recipients of cochlear implant surgery			
			People with congenital immunodeficiencies involving any part of the immune system			
			People with diabetes mellitus			
			People living with HIV			

Province [References]	Pneumococcal vaccination for people aged 65 and older		Pneumococcal vaccination for people aged 18-64 who are at increased risk of IPD			Availability in pharmacies#
	Program start year	Public funding	Groups	Program start year*	Public funding	
			People receiving immunocompromising therapy including long-term systemic corticosteroids, chemotherapy, radiation therapy, post-organ transplant therapy, certain anti-rheumatic drugs.			
			People with chronic liver disease, including hepatitis B and C and hepatic cirrhosis due to any cause			
			People with malignant neoplasms, including leukemia and lymphoma			
			People with chronic renal disease, including nephrotic syndrome			
			People with chronic respiratory disease, excluding asthma unless management requires high dose corticosteroids			
			People with sickle-cell disease or other hemoglobinopathies			
			Solid organ transplant candidates or recipients			
			People with chronic neurologic conditions that may impair clearance of oral secretions			
			HSCT candidates or recipients			
			Residents of nursing homes, homes for the aged and chronic care facilities or wards			
Manitoba [13-15]	2001	Yes	Residents of a personal care home or long-term care facility	2001	Yes	Yes
			People with CSF leak			
			People with cochlear implants			
			People with cardiac or pulmonary disease			
			People with diabetes mellitus			
			People with chronic kidney disease			
			People with liver disease (including hepatic cirrhosis due to any cause)			
			People with hemoglobinopathies			
Saskatchewan [16, 17]	1998	Yes	Residents of extended or intermediate care facilities including group homes	1998	Yes	Yes
			People with alcoholism			
			People with cardiac disease			
			People with CSF disorders			
			Candidates or recipients of a cochlear implant			
			People with cystic fibrosis			
			People with diabetes mellitus			
			Homeless people			
			People with neurological conditions that impede clearance of respiratory/oral secretions			
			People with pulmonary disease (excluding asthma, unless on high dose corticosteroid therapy)			
			People with acquired complement deficiency			
			People with congenital, acquired or functional asplenia			
			People with congenital immunodeficiencies			
			HSCT recipients			
			People living with HIV			
			People receiving immunosuppressive medical treatment (e.g. chemotherapy, radiation, high dose steroids)			
			People with liver disease (including cirrhosis, hepatitis B and hepatitis C)			
			People with malignancies			
			People with renal disease			
			People with sickle-cell disease or other hemoglobinopathies			

Province [References]	Pneumococcal vaccination for people aged 65 and older		Pneumococcal vaccination for people aged 18-64 who are at increased risk of IPD			Availability in pharmacies#
	Program start year	Public funding	Groups	Program start year*	Public funding	
			Solid organ or islet cell transplant candidates or recipients			
Alberta [18-20]	1998	Yes	People with alcoholism	1997	Yes	Yes
			People with functional or anatomic asplenia/hyposplenism			
			People with chronic cardiac disease			
			People with chronic CSF leak			
			People with chronic liver disease, including hepatic cirrhosis due to any cause, hepatitis B carriers and hepatitis C infection			
			People with chronic neurologic conditions that may impair clearance of oral secretions			
			People with chronic pulmonary disease (including asthma requiring medical treatment within the last 12 months regardless of whether they are on high dose steroids)			
			People with chronic renal disease, including nephrotic syndrome			
			Candidates and recipients of cochlear implant surgery			
			People with congenital immunodeficiencies involving any part of the immune system			
			People with diabetes			
			HSCT candidates and recipients			
			Solid organ or islet transplant candidates and recipients			
			People with diabetes mellitus			
			People undergoing or anticipating immunosuppressive therapy			
			People with malignant hematologic disorders (affecting the bone marrow or lymphatic system) including leukemia, lymphoma, Hodgkin's disease and non-Hodgkin's lymphomas, and multiple myeloma			
			People with malignant solid organ tumors either currently or within past 5 years			
			People with sickle-cell disease or other hemoglobinopathies			
			Homeless people and those living in chronically disadvantaged conditions			
			Illicit drug users			
			People receiving treatment with complement-inhibitors			
British Columbia [21, 22]	1998	Yes	HSCT recipients and people with functional or anatomic asplenia	1996	Yes	Yes
			Residents of extended and intermediate care facilities			
			People with CMCs that increase the risk of IPD			
			Solid organ transplant recipients and candidates			
			People with hepatitis C infection			
			Islet cell transplant recipients and candidates			
			People with cystic fibrosis			
			People with asthma, unless management involves high dose oral corticosteroid treatment			
			Homeless and/or illicit drug users			

Abbreviations: CMC, Chronic medical condition; CSF, Cerebrospinal fluid; HIV, Human immunodeficiency virus; HSCT, Hematopoietic stem cell transplant; IPD, invasive pneumococcal disease.

* Start year of the program may vary across conditions, as some categories may have been added among those eligible for vaccination over the years.
\# Depending on the province and provider, some categories for whom pneumococcal vaccination is recommended may not be eligible to receive the vaccine at pharmacies. Also, fees may be required for this service, with variable costs across provinces aånd providers.

References

[1] Department of Health and Community Service. Newfoundland and Labrador Immunization Manual. St. John’s, NL, Canada Department of Health and Community Services; 2019.
[2] Department of Health and Community Services. Pharmacists Assuming Key Role in Newfoundland and Labrador’s Influenza Vaccination Program. St. John’s, NL, Canada Department of Health and Community Services; 2020.
[3] Department of Health and Wellness Chief Public Health Office. Prince Edward Island Adult Immunization Detailed Schedule. Charlottetown, PEI, Canada Department of Health and Wellness; 2019.
[4] Immunizations and Injections. Dartmouth, NS, Canada: Pharmacy Association of Nova Scotia; 2022.
[5] Office of the Chief Medical Officer of Health. Publicly Funded Vaccine Eligibility for Individuals at High Risk of Acquiring Vaccine Preventable Diseases. Halifax, NS, Canada: Department of Health and Wellness; 2019.
[6] Government of Nova Scotia. Nova Scotia Immunization Manual. Nova Scotia: Government of Nova Scotia; 2019.
[7] Folkins C, Brewster C, Foster R, Gulliver A, Thomson E, Gorman-Asal M, et al. Projected outcomes of an expanded role for pharmacy professionals in the provision of publicly funded immunization services in New Brunswick. Fredericton, NB, Canada: New Brunswick Institute for Research, Data and Training; 2021.
[8] Association Pulmonaire du Québec. Vaccination. 2022.
[9] Douville-Fradet M., Amini R., Ouakki M., Deceuninck G., Boulianne N., Lefebvre B., et al. Impact du programme d’immunisation contre les maladies invasives à pneumocoque au Québec, 2010-2014. Institut National de Santé Publique du Québec; 2017.
[10] City of Toronto. Pneumococcal Vaccines Information for Health Professionals. Toronto, ON, Canada: City of Toronto; 2020.
[11] Ontario College of Pharmacists. Pharmacists not authorized to administer additional vaccines. Pharmacy Connection: Ontario College of Pharmacists; 2017.
[12] Public Health Division. Pneumococcal conjugate 13-valent vaccine (Prevnar®13) for adults with high risk medical conditions: Q&A for health care providers Queen's Printer for Ontario; 2014.
[13] Mahmud SM, Sinnock H, Mostaco-Guidolin LC, Pabla G, Wierzbowski AK, Bozat-Emre S. Long-term trends in invasive pneumococcal disease in Manitoba, Canada. Hum Vaccin Immunother. 2017;13:1884-91.
[14] Manitoba Public Health. Manitoba’s Pneumococcal Immunization Program: Frequently Asked Questions & Answers for Health Care Providers. Winnipeg, MB, Canada: Manitoba Public Health; 2019.
[15] Pharmacist Administration of Vaccines in Manitoba. In: Manitoba Po, editor. Winnipeg, MB, Canada: Department of Health, Healthy Living and Seniors; 2014.
[16] Government of Saskatchewan. Saskatchewan Immunization Manual. Regina, SK, Canada: Ministry of Health, Government of Saskatchewan; 2016.
[17] Saskatchewan College of Pharmacy Professionals. Disease Prevention and Travel Health Services Policy and Framework for Saskatchewan Pharmacists. Regina, SK, Canada: Saskatchewan College of Pharmacy Professionals; 2021.
[18] Government of Alberta. Pneumococcal Polysaccharide 23-Valent Immunization Program Edmonton, AB, Canada: Government of Alberta; 2018.
[19] Government of Alberta. Alberta Immunization Policy. Alberta Immunization Program History. Edmonton, ON, Canada: Government of Alberta; 2022.
[20] Province-wide Immunization Program Standards and Quality. Pneumococcal Polysaccharide Vaccine Biological Page. Edmonton, AB, Canada: Alberta Health Services; 2018.
[21] BC Center for Disease Control. Communicable Disease Control Manual. Vancouver, BC, Canada: BC Centre for Disease Control; 2020.
[22] BC Centre for Disease Control. Pharmacist access to publicly funded vaccine in British Columbia: Implementation guideline. Vancouver, BC, Canada: BC Centre for Disease Control, British Columbia Immunization Committee, Pharmacist and Immunization Working Group; 2012.
