# Supplementary material for: Pneumococcal vaccination uptake and missed opportunities for vaccination among Canadian adults: A cross-sectional analysis of the Canadian Longitudinal Study on Aging (CLSA)
Source: PLoS One. 2022 Oct 14;17(10):e0275923. doi: 10.1371/journal.pone.0275923 (PMC9565727; doi:10.1371/journal.pone.0275923)
Supplement: S2 Table — (PDF) [file pone.0275923.s004.pdf]

S2 Table: Description of study variables. For each variable, we report the corresponding survey question and Canadian Longitudinal Study on Aging (CLSA) variable name, response options offered to participants during the survey, and categorization used for the purpose of this study.

Variable group	Variable	Survey question [CLSA variable name]	Possible responses	Categorization for analysis
Outcome	Self-reported pneumococcal vaccination	Have you had pneumonia shot (pneumococcal vaccination) in your life? [PHB_PCV_COF1, PHB_PCV_TRF1]	Yes	Vaccinated
			No	Unvaccinated
			Don't know/No answer/Refused/Missing	Missing
Sociodemographic characteristics	Sex at birth	What was your sex at birth? [SDC_BTHSEX_COF1, SDC_BTHSEX_TRF1)	Female	Female
			Male	Male
			Don't know/No answer/Refused/Missing	Missing
	Age group	Participant age at beginning of FU1 [AGE_NMBR_COF1, AGE_NMBR_TRF1]	Continuous number	<50
				50-54
				55-64
				65-74
				75-84
				85+
	Race	Cultural/racial background [SDC_DCGT_COM, SDC_DCGT_TRM]	White only	White
			Black only	Other than white
			Korean only	
			Filipino only	
			Japanese only	
			Chinese only	
			South Asian only	
			Southeast Asian only	
			Arab only	
			West Asian only	
			Latin American only	
			Other racial or cultural original (only)	
			Multiple racial or cultural origins	
			Missing	Missing
	Highest education level	Highest level of education [ED_UDR04_COM, ED_UDR04_TRM]	Less than secondary school graduation	Less than secondary school graduation
			Secondary school graduation, no post-secondary education	Secondary school graduation, no post-secondary education
			Some post-secondary education	Some post-secondary education
			Post-secondary degree/diploma	Post-secondary degree/diploma
			Missing	Missing
	Annual household income	What is your best estimate of the total household income received by all household members, from all sources, before taxes and deductions, in the past	Less than $20,000	<$20,000
			$20,000 or more, but less than $50,000	$20,000 to <$50,000
			$50,000 or more, but less than $100,000	$50,000 to <$100,000
			$100,000 or more, but less than $150,000	$100,000 to <$150,000

Variable group	Variable	Survey question [CLSA variable name]	Possible responses	Categorization for analysis
		12 months? [INC_TOT_COF1, INC_TOT_TRF1]	$150,000 or more	$150,000 or higher
			Don't know/No answer/Refused/Missing	Missing
	Marital/partner status	What is your current marital/partner status? [SDC_MRTL_COF1, SDC_MRTL_TRF1]	Single, never married or never lived with a partner	Single/Never married/Never lived with a partner
			Married, living with a partner in a common-law relationship	Married/Common-law
			Widowed	Widowed
			Divorced	Divorced/Separated
			Separated	
			Don't know/No answer/Refused/Missing	Missing
	Province of residence	Province of residence [WGHTS_PROV_COF1, WGHTS_PROV_TRF1]	Newfoundland	Newfoundland
			Prince Edward Island	Prince Edward Island
			Nova Scotia	Nova Scotia
			New Brunswick	New Brunswick
			Quebec	Quebec
			Ontario	Ontario
			Manitoba	Manitoba
			Saskatchewan	Saskatchewan
			Alberta	Alberta
			British Columbia	British Columbia
	Geographic area of residence	Urban/rural classification [SDC_URBAN_RURAL_COF1, SDC_URBAN_RURAL_TRF1]	Rural area	Rural
			Rural fringe in CMAs/CAs	
			Link to DA	
			Urban core	Urban
			Urban fringe	
			Urban areas out CMAs/CAs	
			Secondary urban core	
			Missing	Missing
Variables related to health status and healthcare utilization	At least one chronic medical condition	Cardiovascular disease:<br><br>Has a doctor ever told you that you have…<br>- heart disease (including congestive heart failure or CHF)? [CCC_HEART_COF1, CCT_HEART_TRF1]	Yes/No/Missing	Yes if "yes" to at least one of the relevant questions<br><br>No if "no" to all relevant questions<br><br>Missing if all "missing"
		- heart attack or myocardial infarction? [CCC_AMI_COF1, CCT_AMI_TRF1]	Yes/No/Missing	
			Yes/No/Missing	

Variable group	Variable	Survey question [CLSA variable name]	Possible responses	Categorization for analysis
		- angina (or chest pain due to heart disease)? [CCC_ANGI_COF1, CCT_ANGI_TRF1]		
		- high blood pressure or hypertension? [CCC_HBP_COF1, CCC_HBP_TRF1]	Yes/No/Missing	
		Chronic lung disease:<br><br>Has a doctor ever told you that you have:<br>- any of the following: emphysema, chronic bronchitis, chronic obstructive pulmonary disease (COPD), or chronic changes in lungs due to smoking? [CCC_COPD_COF1, CCT_COPD_TRF1]	Yes/No/Missing	
		- asthma? [CCC_ASTHM_COF1, CCT_ASTHM_TRF1]	Yes/No/Missing	
		Cerebrovascular disease:<br><br>Has a doctor ever told you that you have experienced:<br>- a stroke or cerebrovascular accident (CVA)? [CCC_CVA_COF1, CCT_CVA_TRF1]	Yes/No/Missing	
		- a ministroke or TIA (transient ischemic attack)? [CCC_TIA_COF1, CCT_TIA_TRF1]	Yes/No/Missing	
		Chronic kidney disease:<br><br>Has a doctor ever told you that you have kidney disease or kidney failure? [CCC_KIDN_COF1, CCT_KIDN_TRF1]	Yes/No/Missing	
		Diabetes mellitus:<br><br>Has a doctor ever told you that you have diabetes, borderline diabetes or that your blood sugar is high? [DIA_DIAB_COF1, CCT_DIAB_TRF1]	Yes/No/Missing	

Variable group	Variable	Survey question [CLSA variable name]	Possible responses	Categorization for analysis
		Cancer:<br><br>Has a doctor ever told you that you had cancer? [CCC_CANC_COF1, CCT_CANC_TRF1]	Yes/No/Missing	
		Chronic neurologic condition:<br><br>Has a doctor ever told you that you have…<br>- dementia or Alzheimer's disease? [CCC_ALZH_COF1, CCT_ALZH_TRF1]	Yes/No/Missing	
		- Parkinson's disease? [CCC_PARK_COF1, PKD_PARK_TRF1]	Yes/No/Missing	
		- multiple sclerosis? [CCC_MS_COF1, CCT_MS_TRF1]	Yes/No/Missing	
	Contact with a family doctor in previous 12 months	During the past 12 months, have you had contact with a family doctor? [HCU_FAMPHY_COF1, HCU_FAMPHY_TRF1]	Yes	Yes
			No	No
			Don't know/No answer/Refused/Missing	Missing
	Influenza vaccination in previous 12 months	Have you had a flu shot in the last 12 months? [PHB_FLUV_COF1, PHB_FLUV_TRF1]	Yes	Vaccinated
			No	Unvaccinated
			Don't know/No answer/Refused/Missing	Missing
