# Supplementary material for: Pneumococcal vaccination uptake and missed opportunities for vaccination among Canadian adults: A cross-sectional analysis of the Canadian Longitudinal Study on Aging (CLSA)
Source: PLoS One. 2022 Oct 14;17(10):e0275923. doi: 10.1371/journal.pone.0275923 (PMC9565727; doi:10.1371/journal.pone.0275923)
Supplement: S3 Table — (PDF) [file pone.0275923.s005.pdf]

S3 Table: Distribution of chronic medical conditions (CMC) among individuals at increased risk of invasive pneumococcal disease (IPD) by self-reported pneumococcal vaccination status (vaccinated or unvaccinated during lifetime). Counts, percentages, and 95% confidence intervals within variable strata are shown for two subgroups of interest: 1) individuals aged 65 and older (n = 22,246), and 2) individuals aged 47-64 with at least one chronic medical condition (CMC) among those listed in the table (cardiovascular disease, chronic lung disease, cerebrovascular disease, chronic kidney disease, diabetes mellitus, cancer, chronic neurologic condition) (n = 10,815).

**Characteristic**	**Self-reported pneumococcal vaccination in lifetime**							
	**Individuals aged 65 and older (n = 22,246)**				**Individuals aged < 65 with at least one CMC (n = 10,815)**			
	**Vaccinated**		**Unvaccinated**		**Vaccinated**		**Unvaccinated**	
	N	% (95% CI)	N	% (95% CI)	N	% (95% CI)	N	% (95% CI)
**Overall**	12,054	54.2 (53.5-54.8)	10,192	45.8 (45.2-46.5)	1,989	19.3 (18.6-20.1)	8,301	80.7 (79.9-81.4)
**Chronic medical condition**								
At least one	9,664	57.1 (56.4-57.9)	7,254	42.9 (42.1-43.6)	2.027	100	8,788	100
None	2,095	43.8 (42.4-45.2)	2,687	56.2 (54.8-57.6)	0	NA	NA	NA
Missing	295	54.0 (49.8-58.2)	251	45.7 (41.8-50.,2)	0	NA	NA	NA
**Cardiovascular disease**								
No	4,400	48.7 (47.6-49.7)	4,640	51.3 (50.3-52.4)	794	17.6 (16.5-18.7)	3,723	82.4 (81.3-83.5)
Yes	7,375	58.1 (57.3-59.0)	5,316	41.9 (41.0-42.7)	1,233	19.6 (18.6-20.6)	5,065	80.4 (79.4-81.4)
Missing	279	54.2 (49.9-58.4)	236	45.8 (41.6-50.1)	0	0	0	0
**Chronic lung disease**								
No	9,212	51.5 (50.8-52.3)	8,664	48.5 (47.7-49.2)	1,110	15.2 (14.4-16.1)	6,181	84.8 (83.9-85.6)
Yes	2,554	66.6 (65.1-68.0)	1,283	33.4 (32.0-34.9)	912	25.9 (24.5-27.4)	2,605	74.1 (72.6-75.5)
Missing	288	54.0 (49.8-58.2)	245	46.0 (41.8-50.2)	5	71.4 (32.7-92.8)	2	28.6 (7.2-67.3)
**Cerebrovascular disease**								
No	10,695	53.8 (53.1-54.4)	9,202	46.2 (45.6-46.9)	1,911	18.5 (17.7-19.2)	8,442	81.5 (80.8-82.3)
Yes	1,057	59.1 (56.8-58.0)	732	40.9 (38.7-43.2)	115	25.2 (21.4-29.4)	341	74.8 (70.6-78.6)
Missing	302	54.2 (53.5-54.8)	258	46.1 (42.0-50.2)	1	16.7 (2.3-63.1)	5	83.3 (36.9-97.7)
**Chronic kidney disease**								
No	11,119	53.7 (53.0-54.4)	9,581	46.3 (45.6-47.0)	1,915	18.4 (17.7-19.2)	8,467	81.6 (80.8-82.3)
Yes	620	64.0 (60.9-66.9)	349	36.0 (33.1-39.1)	109	25.8 (21.9-30.2)	313	74.2 (69.8-78.1)
Missing	315	54.6 (50.5-58.6)	262	45.4 (41.4-49.5)	3	27.3 (9.0-58.6)	8	72.7 (41.4-91.0)
**Diabetes mellitus**								
No	8,861	52.9 (52.1-53.6)	7,902	47.1 (46.4-47.9)	1,218	16.0 (15.2-16.9)	6,390	84.0 (83.1-84.8)
Yes	2,888	58.3 (57.3-60.0)	2,036	41.3 (40.0-42.7)	804	25.2 (23.7-26.7)	2.392	74.8 (73.3-76.3)
Missing	305	54.6 (50.4-58.6)	254	45.4 (41.4-49.6)	5	45.5 (20.3-73.2)	6	54.5 (26.8-79.7)
**Cancer**								
No	8,455	52.5 (51.8-53.3)	7,635	47.5 (46.7-48.2)	1,582	18.3 (17.52-19.1)	7,054	81.7 (80.9-82.5)
Yes	3,306	59.0 (57.7-60.2)	2,301	41.0 (39.8-42.3)	442	20.4 (18.7-22.1)	1,727	79.6 (77.9-81.3)
Missing	293	53.4 (49.2-57.5)	256	46.6 (42.5-50.8)	3	30.0 (10.0-62.4)	7	70.0 (37.6-90.0)
**Chronic neurologic condition**								
No	11,532	54.0 (53.4-54.7)	9,805	46.0 (45.3-46.6)	1,966	18.6 (17.9-19.3)	8.611	81.4 (80.7-82.1)
Yes	243	61.5 (56.6-66.2)	152	38.5 (33.8-43.4)	61	25.6 (20.5-31.6)	177	74.4 (68.4-79.5)
Missing	279	54.3 (50.0-58.5)	235	45.7 (41.5-50.0)	0	0	0	0

Abbreviations: CI, confidence interval; CMC, chronic medical condition; NA, not applicable.
