# Supplementary material for: Pneumococcal vaccination uptake and missed opportunities for vaccination among Canadian adults: A cross-sectional analysis of the Canadian Longitudinal Study on Aging (CLSA)
Source: PLoS One. 2022 Oct 14;17(10):e0275923. doi: 10.1371/journal.pone.0275923 (PMC9565727; doi:10.1371/journal.pone.0275923)
Supplement: S4 Table — (PDF) [file pone.0275923.s006.pdf]

S4 Table: Results of sensitivity analyses of factors associated with under-vaccination for pneumococcal disease among 19,742 Canadian Longitudinal Study on Aging (CLSA) cohort participants aged 65 and older who had complete data on all variables of interest – Scenarios 1-4. Adjusted odds ratios (aORs) and 95% confidence intervals (CIs) were estimated from multivariable logistic regression models. For sensitivity analysis, we simulated the following scenarios to evaluate the impact of the potential misclassification of participants' pneumococcal vaccination status on estimated associations: i) sensitivity (Se) of 70% (i.e., 30% of those who reported being vaccinated were in fact unvaccinated) and specificity (Sp) of 80% (i.e., 80% of those who reported being unvaccinated were truly unvaccinated) (Scenario 1); ii) Se = 75% and Sp = 85% (Scenario 2); iii) Se = 80% and Sp = 90% (Scenario 3); iv) Se = 85% and Sp = 95% (Scenario 4). Results of the primary analysis, based on the actual survey responses, are provided for comparison.

**Variable**	**Primary analysis**	**Sensitivity analyses**			
		**Scenario 1 (Se=70%, Sp=80%)**	**Scenario 2 (Se=75%, Sp=85%)**	**Scenario 3 (Se=80%, Sp=90%)**	**Scenario 4 (Se=85%, Sp=95%)**
	aOR (95% CI)	*aOR (95% CI)*	*aOR (95% CI)*	*aOR (95% CI)*	*aOR (95% CI)*
**Sex at birth[a]**					
Female	Reference	Reference	Reference	Reference	Reference
Male	1.34 (1.26-1.42)	1.17 (1.10-1.24)	1.16 (1.09-1.23)	1.23 (1.16-1.30)	1.24 (1.17-1.32)
**Age group (years)[a]**					
65-74	Reference	Reference	Reference	Reference	Reference
75-84	0.53 (0.49-0.56)	0.73 (0.69-0.78)	0.71 (0.67-0.76)	0.65 (0.61-0.70)	0.62 (0.58-0.66)
≥85	0.46 (0.41-0.52)	0.63 (0.56-0.71)	0.62 (0.55-0.69)	0.59 (0.52-0.66)	0.58 (0.52-0.66)
**Race[a]**					
White	Reference	Reference	Reference	Reference	Reference
Other than white	1.23 (1.05-1.44)	1.08 (0.93-1.26)	1.28 (1.09-1.49)	1.12 (0.96-1.31)	1.20 (1.03-1.40)
**Highest education level[a]**					
Less than secondary school education	Reference	Reference	Reference	Reference	Reference
Secondary school grad., no post-secondary educ.	1.00 (0.87-1.14)	0.89 (0.78-1.01)	0.99 (0.87-1.13)	0.96 (0.84-1.10)	0.93 (0.82-1.07)
Some post-secondary education	0.95 (0.82-1.11)	0.95 (0.82-1.10)	0.94 (0.81-1.09)	0.88 (0.76-1.02)	0.92 (0.79-1.06)
Post-secondary degree/diploma	0.92 (0.82-1.03)	0.91 (0.82-1.02)	0.95 (0.85-1.06)	0.93 (0.83-1.04)	0.87 (0.78-0.98)
**Annual household income (in Canadian dollars)[a]**					
Less than $20,000	Reference	Reference	Reference	Reference	Reference
$20,000 to <$50,000	0.88 (0.77-1.00)	0.97 (0.85-1.10)	0.91 (0.80-1.04)	0.99 (0.87-1.12)	0.89 (0.78-1.01)
$50,000 to <$100,000	0.72 (0.63-0.83)	0.90 (0.79-1.03)	0.81 (0.71-0.93)	0.83 (0.73-0.96)	0.78 (0.68-0.90)
$100,000 to <$150,000	0.67 (0.57-0.78)	0.91 (0.78-1.06)	0.84 (0.72-0.98)	0.78 (0.67-0.91)	0.70 (0.60-0.82)
$150,000 or higher	0.72 (0.61-0.86)	0.90 (0.76-1.07)	0.84 (0.71-1.00)	0.84 (0.71-1.00)	0.81 (0.68-0.97)
**Marital/partner status[a]**					
Single/Never married/Never lived with a partner	Reference	Reference	Reference	Reference	Reference
Married/Common-law	0.90 (0.80-1.02)	0.92 (0.81-1.04)	0.85 (0.76-0.96)	0.92 (0.81-1.04)	0.91 (0.81-1.03)
Widowed	0.80 (0.70-0.92)	0.90 (0.78-1.02)	0.78 (0.68-0.89)	0.82 (0.72-0.94)	0.79 (0.69-0.91)
Divorced/Separated	1.17 (1.02-1.34)	1.10 (0.95-1.26)	1.03 (0.89-1.18)	1.14 (1.00-1.31)	1.14 (0.99-1.31)
**Province of residence[a]**					
Ontario	Reference	Reference	Reference	Reference	Reference
Newfoundland	2.33 (2.04-2.66)	1.40 (1.23-1.59)	1.72 (1.51-1.97)	1.69 (1.48-1.92)	2.07 (1.81-2.37)
Prince Edward Island	1.48 (1.21-1.81)	1.08 (0.89-1.32)	1.32 (1.08-1.62)	1.24 (1.01-1.52)	1.42 (1.15-1.74)
Nova Scotia	1.06 (0.94-1.20)	1.00 (0.90-1.13)	1.10 (0.98-1.24)	0.91 (0.81-1.03)	1.03 (0.92-1.16)
New Brunswick	1.49 (1.21-1.82)	1.43 (1.17-1.75)	1.47 (1.20-1.79)	1.20 (0.98-1.47)	1.42 (1.16-1.74)
Quebec	0.74 (0.67-0.81)	0.84 (0.77-0.92)	0.88 (0.81-0.97)	0.76 (0.69-0.83)	0.81 (0.74-0.89)
Manitoba	0.67 (0.60-0.76)	0.82 (0.73-0.92)	0.86 (0.76-0.96)	0.73 (0.65-0.82)	0.75 (0.67-0.84)
Saskatchewan	0.86 (0.71-1.06)	0.90 (0.74-1.09)	0.91 (0.75-1.11)	0.81 (0.67-0.99)	0.96 (0.79-1.17)
Alberta	0.68 (0.61-0.76)	0.78 (0.70-0.86)	0.83 (0.74-0.92)	0.70 (0.63-0.78)	0.73 (0.66-0.82)

Variable	Primary analysis	Sensitivity analyses			
		**Scenario 1 (Se=70%, Sp=80%)**	**Scenario 2 (Se=75%, Sp=85%)**	**Scenario 3 (Se=80%, Sp=90%)**	**Scenario 4 (Se=85%, Sp=95%)**
	aOR (95% CI)	*aOR (95% CI)*	*aOR (95% CI)*	*aOR (95% CI)*	*aOR (95% CI)*
British Columbia	1.03 (0.94-1.13)	0.99 (0.91-1.08)	1.11 (1.01-1.21)	0.99 (0.90-1.08)	1.04 (0.95-1.14)
**Urbanicity of residence**					
Urban	Reference	Reference	Reference	Reference	Reference
Rural	1.13 (1.0-1.2)	1.07 (0.98-1.16)	1.12 (1.03-1.22)	1.09 (1.01-1.19)	1.10 (1.01-1.20)
**At least one CMC[b]**					
No	Reference	Reference	Reference	Reference	Reference
Yes	0.70 (0.65-0.75)	0.88 (0.81-0.94)	0.84 (0.78-0.90)	0.81 (0.75-0.87)	0.75 (0.69-0.80)
**Contact with a family doctor in prior 12 months[b]**					
No	Reference	Reference	Reference	Reference	Reference
Yes	0.73 (0.64-0.84)	0.89 (0.78-1.02)	0.88 (0.77-1.00)	0.77 (0.67-0.88)	0.78 (0.67-0.89)
**Influenza vaccination in prior 12 months[b]**					
No	Reference	Reference	Reference	Reference	Reference
Yes	0.14 (0.13-0.15)	0.41 (0.38-0.44)	0.34 (0.31-0.36)	0.27 (0.25-0.29)	0.21 (0.19-0.36)

Abbreviations: aOR, adjusted odds ratio; CI, confidence interval; CMC, chronic medical condition; Se, sensitivity; Sp, specificity.

[a] Estimated from a model including all sociodemographic variables (sex at birth, age group, race, highest education level, annual household income, province of residence, and urbanicity) categorized as shown in the table.
[b] Estimated from a fully adjusted model including all variables listed in the table.
